# Hierarchical Post-transcriptional Regulation of Colicin E2 Expression in *Escherichia coli*

**DOI:** 10.1371/journal.pcbi.1005243

**Published:** 2016-12-15

**Authors:** Matthias Lechner, Mathias Schwarz, Madeleine Opitz, Erwin Frey

**Affiliations:** 1 Arnold-Sommerfeld-Center for Theoretical Physics and Center for NanoScience, Ludwig-Maximilians-Universität, Munich, Germany; 2 Institute for Biological and Medical Imaging, Technische Universität München and Helmholtz Zentrum München, Neuherberg, Germany; Rice University, UNITED STATES

## Abstract

Post-transcriptional regulation of gene expression plays a crucial role in many bacterial pathways. In particular, the translation of mRNA can be regulated by trans-acting, small, non-coding RNAs (sRNAs) or mRNA-binding proteins, each of which has been successfully treated theoretically using two-component models. An important system that includes a combination of these modes of post-transcriptional regulation is the Colicin E2 system. DNA damage, by triggering the SOS response, leads to the heterogeneous expression of the Colicin E2 operon including the *cea* gene encoding the toxin colicin E2, and the *cel* gene that codes for the induction of cell lysis and release of colicin. Although previous studies have uncovered the system’s basic regulatory interactions, its dynamical behavior is still unknown. Here, we develop a simple, yet comprehensive, mathematical model of the colicin E2 regulatory network, and study its dynamics. Its post-transcriptional regulation can be reduced to three hierarchically ordered components: the mRNA including the *cel* gene, the mRNA-binding protein CsrA, and an effective sRNA that regulates CsrA. We demonstrate that the stationary state of this system exhibits a pronounced threshold in the abundance of free mRNA. As post-transcriptional regulation is known to be noisy, we performed a detailed stochastic analysis, and found fluctuations to be largest at production rates close to the threshold. The magnitude of fluctuations can be tuned by the rate of production of the sRNA. To study the dynamics in response to an SOS signal, we incorporated the LexA-RecA SOS response network into our model. We found that CsrA regulation filtered out short-lived activation peaks and caused a delay in lysis gene expression for prolonged SOS signals, which is also seen in experiments. Moreover, we showed that a stochastic SOS signal creates a broad lysis time distribution. Our model thus theoretically describes Colicin E2 expression dynamics in detail and reveals the importance of the specific regulatory components for the timing of toxin release.

## Author Summary

Gene expression is a fundamental biological process, in which living cells use genetic information to synthesize functional products like proteins. To control this process, cells make use of many different mechanisms. A well-studied example is the binding of expression intermediates by a cellular component in order to delay the synthesis. This mechanism is known to regulate the stress-induced release of the toxin colicin E2 by *E. coli* bacteria. However, experimental studies have shown that this system is not regulated by just one component, but the interplay of several cellular components, in which the hierarchically ordered main components interact. Here, we create a mathematical model for the interaction network of colicin E2 release, and study how the component levels evolve. We show that the system is able to delay the release of the toxin. Additional components allow to fine-tune the delay and dampen fluctuations in gene expression that would lead to premature toxin release. A comprehensive analysis of the time evolution reveals a broad distribution of toxin release times, which is also observed in experiments. This rich dynamical behavior emerges from the interplay of regulatory components, and, due to its generality, may also be transferred to similar regulatory networks, in particular toxin expression systems.

## Introduction

Regulation of gene expression occurs at transcriptional and post-transcriptional levels, and has been studied intensively both experimentally and theoretically [1–10]. Bacterial stress responses, such as the well-studied production and release of the toxin colicin E2 in *Escherichia coli*, represent one setting in which post-transcriptional control is crucial [11–15].

**Fig 1:**
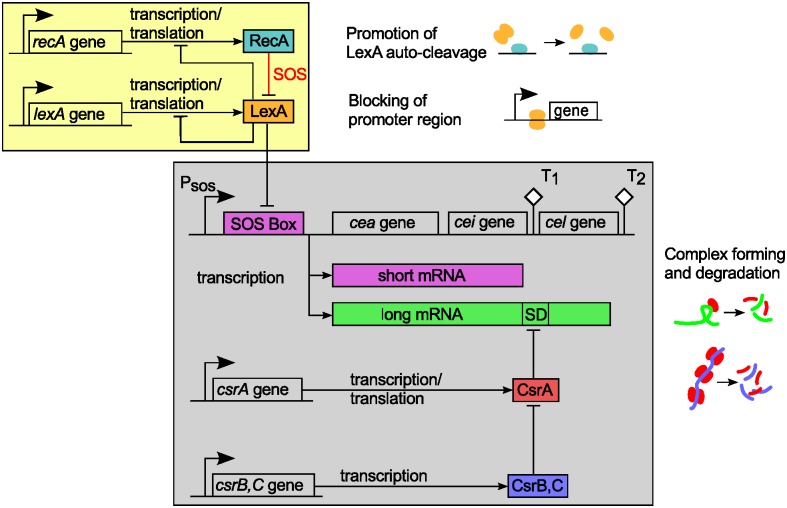
Regulation of colicin E2 expression and release. The interaction scheme is a generalized adaption of that presented by Yang [28]. Under normal conditions, the SOS response system (yellow box) maintains a constant level of LexA dimers, which repress the SOS promoter of the colicin E2 system (gray box). In the event of DNA damage, RecA is activated and promotes auto-cleavage of LexA. This permits the transcription of two different mRNAs: Short mRNA codes for components of colicin immunity complexes (colicin gene *cea*, immunity gene *cei*), whereas long mRNA additionally encodes the protein that triggers cell lysis. Translation of long mRNA is regulated by binding of the protein CsrA to its Shine-Dalgarno sequence (SD). CsrA itself is regulated by the two sRNAs CsrB and CsrC. Other elements: *P*_sos_: SOS promoter; *T*_1_ and *T*_2_: transcriptional terminators.

Colicins are toxic proteins produced by certain *E. coli* strains in response to stress as a means to kill bacteria that compete with them for the same resources. More specificly, colicin E2 is a bacteriocin, which damages the DNA of bacterial cells that absorb it (a DNAse). Once synthesized, colicin E2 forms a complex with an immunity protein, thus protecting its producer from its otherwise lethal action [14, 16, 17]. The toxin is released only upon cell lysis, which is triggered by the synthesis of a dedicated lysis protein [15, 18–20]. As this inevitably entails the death of the producer cell [19], it is vital for the persistence of the population that only a fraction of its members actually releases the toxin [14]. The genes for the colicin, immunity protein and lysis protein are organized into the colicin E2 operon, which is depicted in Fig 1, together with the interaction network that controls colicin E2 expression and release.

Each of the three components is encoded by a single gene—the colicin by *cea*, the colicin-specific immunity protein by *cei*, and the lysis factor by *cel*—and three regulatory regions control their transcription: an SOS promoter upstream of the *cea* gene [21], and two transcriptional terminators *T*_1_ and *T*_2_, located upstream and downstream of the *cel* gene, respectively [22]. The key transcriptional regulator of the SOS operon is the LexA protein (reviewed in [23]), marked in orange in Fig 1. LexA dimers repress the SOS promoter region of the ColE2 operon, but also block the transcription of over 30 other SOS genes [24, 25], many of which play an important role in DNA repair [26]. In the event of DNA damage, the LexA dimer undergoes auto-cleavage upon interaction with RecA [27], and the transcription of SOS genes begins. The presence of the two transcriptional terminators in the ColE2 operon results in the production of two different mRNAs: A shorter transcript (short mRNA, marked purple in Fig 1) that encompasses only the genes for the toxin colicin E2 and the immunity protein, and a longer transcript (long mRNA, marked green in Fig 1), which additionally includes the lysis gene [14, 28, 29–32]. Hence, lysis can only be initiated after the translation of long mRNA [18], and this crucial operation is regulated at the post-transcriptional level, as described below.

Post-transcriptional regulation makes use of many different mechanisms. Recent studies emphasize the particular importance of non-coding sRNAs [33] for various processes in *E. coli*, especially because of their ability to introduce delays and set up thresholds for translation [34–37]. This is done either directly, by sRNAs pairing with their target mRNA (sRNA-mRNA interaction), or indirectly, by sequestering of specific mRNA-binding proteins (mRNA-protein interaction) [2, 38, 39]. For the latter form of regulation, recent studies highlighted the importance of the production rates of regulatory components [40]. In the case of the ColE2 system, the translation of the long mRNA is regulated by the carbon storage regulator protein CsrA [28], marked red in Fig 1. CsrA dimers destabilize target mRNAs by binding to a region that includes the ribosome-binding site (Shine-Dalgarno sequence) [41]. Masking of the ribosome-binding site by CsrA thus not only represses translation of the lysis gene but also promotes degradation of the long mRNA. However, CsrA is also recognized by two specific sRNAs, CsrB and CsrC [42], marked blue in Fig 1. These sRNAs can therefore sequester CsrA dimers, preventing them from binding to target mRNAs [43–45]. Thus, translation of the ColE2 lysis gene is indirectly regulated by sequestration of CsrA. This process, also known as “molecular titration”, exhibits ultrasensitive thresholds and has been extensively studied [46, 47].

**Fig 2:**
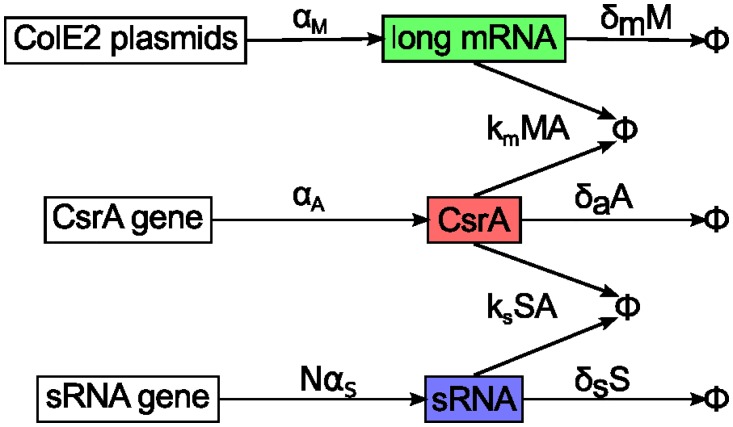
Simplified interaction scheme for post-transcriptional regulation of long mRNA. M,A,S: molecule numbers of free long mRNA, free CsrA dimers and the free effective sRNA; *α*: production rates; *δ*: degradation rates; *k*: effective rate of coupled degradation. The interaction network (see S1 Fig) of the regulatory system depicted in Fig 1 was reduced to a three component system. In both figures, the corresponding components have the same colors. In particular, we combined the complex dynamics (binding, dissociation, degradation) into an effective coupled degradation. The dynamics of sRNA complexes with N binding sites for CsrA and production rate *α*_*S*_ were simplified to the dynamics of an effective sRNA with one CsrA binding site but N-times higher production rate (S1 Text).

The basic interaction network that controls the ColE2 regulatory network has been studied in great detail in previous works [48–51], and many of its functional characteristics, in particular the threshold behavior, were described for a wide range of both bacterial and eukaryotic systems [52]. However, a detailed theoretical description of the dynamics leading to the release of colicin is still missing, in particular the role of the hierarchically ordered regulation involving CsrB and CsrC. In this work, we have formulated this post-transcriptional network in a detailed mathematical model, constructed by analogy to studies of simpler sRNA-regulated systems (for example, [33, 34, 36]). We then simplified the model by assuming fast complex equilibration, and combining the sRNAs CsrB and CsrC into a single, effective sRNA (see S1 Text for details). This reduced the regulation network to three relevant components: free long mRNA, free CsrA and the effective sRNA (see Fig 2). We then analyzed this simplified network in detail. In contrast to previous work [36], we give a general analytical solution for the three component system, and derive a precise approximation for fast and clear analysis. This analytic solution exhibits a pronounced threshold in mRNA production due to CsrA-dependent regulation, which was also confirmed using numeric simulations. We investigated, how this threshold depends on system parameters, and how it affects the actual biological system. Furthermore, we have analyzed the role of fluctuations in the post-transcriptional regulation network and how fluctuations in long mRNA expression may be dampened by sRNA. Finally, we extended our model by including the transcriptional regulation, and analyzed how the system behaves during a realistic SOS response. Previous studies have shown discrete activation peaks in LexA-repressed promoters [26] that can lead to large fluctuations close to the threshold of mRNA expression [9]. In a stochastic simulation of the complete model, we were able to reproduce this phenomenon. Comparison with experimental data on lysis time distributions [48] also shows that our model can explain the delayed and broadly distributed release times of colicin complexes. This underlines the importance of stochasticity for the heterogeneous expression of colicin E2 in *E. coli* populations.

## Results

### A mathematical model for post-transcriptional regulation of colicin E2 release

For our theoretical analysis, we initially developed a detailed mathematical model for the post-transcriptional regulation of colicin E2 release. To this end, we derived a set of coupled, deterministic rate equations from the interaction network depicted in Fig 1, with the corresponding rates for transcription, degradation, binding interactions etc. as parameters. In the following, we briefly review how we reduced the network to its core components, which comprise the theoretical model. The interaction scheme underlying the complete model is presented in S1 Fig and further explanations can be found in the Supporting Information, where we also detail how our model can account for sequestration by other targets of the global regulator CsrA.

As we wished to study the post-transcriptional regulation of colicin E2 expression, we included in the model only those components that are relevant at that stage. The model therefore omits the short mRNA and its products. However, the rate of transcription of the long mRNA is a crucial parameter, which is influenced by the kinetics of activation of the SOS promoter, and thus by the processing of its repressor LexA. Upon DNA damage, RecA promotes auto-cleavage of LexA dimers, thus removing inhibition of the SOS response (marked in red in Fig 1). The LexA-RecA interaction network has recently been modeled stochastically [53]. Before including this detailed network in our final model, we focused on understanding the post-transcriptional dynamics. To this end, we initially assumed that activation of the SOS promoter occurs rapidly relative to the rates of production and degradation of the long mRNA [54], which allowed us to approximate the transcription rate of long mRNA by an effective rate *α*_*M*_ (Materials and Methods). With respect to post-transcriptionally relevant components, we were then left with long mRNA, CsrA, and the two sRNAs CsrB and CsrC, and the mRNA-CsrA-, CsrA-CsrB-, and CsrA-CsrC-complexes.

CsrB and CsrC regulate CsrA by forming complexes with it. The two sRNAs each have several (on average: *N*) CsrA binding sites, and if every occupation state of the sRNAs were to be modeled as a separate component, a large number of coupled rate equations would need to be added to the model. However, due to the fast dynamics of the CsrA-CsrB- and CsrA-CsrC-complexes, and their virtually identical biochemical behavior, we were able to reduce the sRNA interaction to a single equation for an effective sRNA, with only one binding site and transcription rate *Nα*_*S*_ (see Materials and Methods). As a result, the mechanisms of complex formation, dissociation and degradation are replaced by an effective coupled degradation of complex partners. Despite the different processes that are integrated to effective ones, the effective sRNA still resembles the dynamical behavior of CsrB/CsrC. A detailed derivation of the simplified system of rate equations can be found in S1 Text. The final post-transcriptional model is thus reduced to a set of three coupled, deterministic rate equations that capture the behavior of the free long mRNA (*M*), free CsrA dimers (*A*), and an effective free sRNA (*S*) component with a single CsrA binding site:

$$\dot{M}=\alpha_{M}-\delta_{M}M-k_{M}MA,$$ (1)

$$\dot{A}=\alpha_{A}-\delta_{A}A-k_{M}p_{M}MA-k_{S}p_{S}AS,$$ (2)

$$\dot{S}=N\alpha_{S}-\delta_{S}S-Ak_{S}S,$$ (3)

where (1 − *p*_*M*_) and (1 − *p*_*S*_) are the probabilities for CsrA to survive the coupled degradation. A graphical illustration of this differential equation system is depicted in Fig 2. Note that in the model the quantities *M*, *A* and *S* represent the abundance of the corresponding *free* components. Once a long mRNA, sRNA, or CsrA dimer binds to some other component, it loses its function and is thus removed from the model system.

For the analysis of our model, we had to determine production, degradation and binding rates. The particular values used are listed in S1 Table. As far as possible, we chose values that are measured in studies on either the same or comparable systems (see S1 Text for details). In the other cases, we tried to derive plausible parameters from known factors that influence the particular rate. A detailed motivation and derivation of these rates is given in chapter 2 of S1 Text.

### Post-transcriptional regulation yields a tunable rate threshold in mRNA abundance

**Fig 3:**
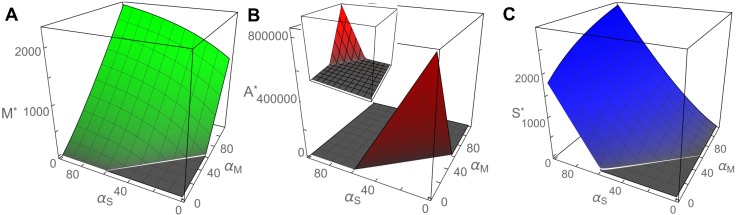
Approximate stationary solutions for (A) long mRNA, (B) CsrA dimers and (C) sRNA. The stationary solutions are given as a function of the effective transcription rate *α*_*M*_ of long mRNA and the production rate *α*_*S*_ of sRNA. The production rate of CsrA dimers was set to *α*_*A*_ = 58.52. All other system parameters are given in S1 Table. For values of *α*_*M*_ and *α*_*S*_ below the threshold, the abundances of free long mRNA and sRNA are zero, as any newly produced component quickly forms a complex with the highly abundant CsrA. At sufficiently large production or transcription rates, sRNA and long mRNA titrate all available CsrA molecules and can thus attain non-zero molecule numbers, The white line gives the transition between two approximate analytical solutions (Materials and Methods).

We analyzed the reduced post-transcriptional model by first calculating its steady state. In order to obtain a cleaner and simpler result, we derived an approximation (see Materials and Methods) for the steady state solution, which agreed very well with the results of numerical simulations (see S2 Fig). Using these simplified equations, we then investigated the impact of the rates of production of long mRNA (*α*_*M*_) and sRNA (*α*_*S*_) on the levels of the three components. The results (see Fig 3) reveal a linear threshold that appears at the same position for all three components. The threshold divides the parameter space into two regimes, in which either CsrA or long mRNA and sRNA have a non-zero abundance. This is due to the coupling between the degradation of CsrA and the abundance of both long mRNA and sRNA, such that the presence of CsrA dimers excludes that of long mRNA and sRNA, and *vice versa*. This mechanism in turn controls the release of colicin-immunity complexes, since a sufficiency of CsrA dimers ensures reliable repression of the long mRNA and prevents synthesis of the lysis protein.

**Fig 4:**
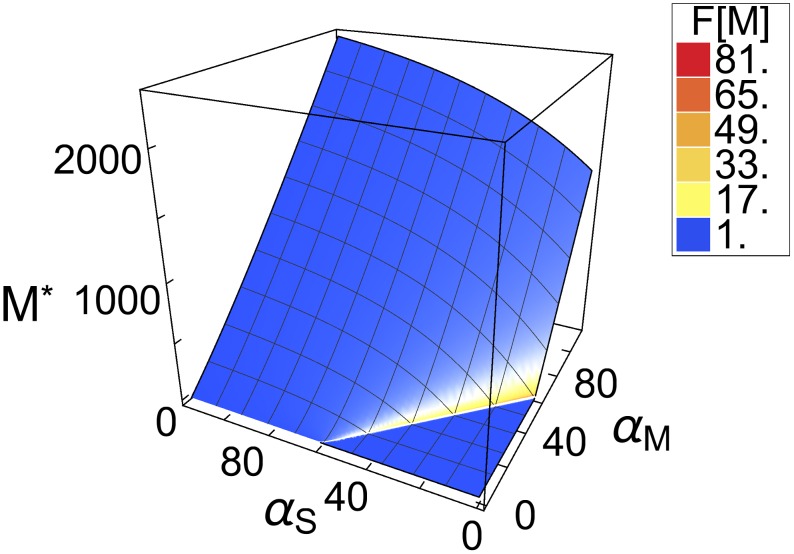
Fluctuations in long mRNA abundance. The fluctuations are quantified by the Fano factor (see main text) and depicted as heatmap in the plot. They are most pronounced at the threshold, and fade for parameter sets above the threshold. With an increase in sRNA production (*Nα*_*S*_), the fluctuations become smaller and more localized to the threshold. This illustrates how the third component sRNA acts as a means to reduce intrinsic fluctuations. The production rate of CsrA dimers was again set to *α*_*A*_ = 58.52, and all other system parameters are given in S1 Table.

From the aforementioned analytic solution we calculated the threshold position as a function of the system parameters (S1 Text). We found that the threshold for non-zero levels of long mRNA lies exactly at the point where the production rate of CsrA *α*_*A*_ is equal to the sum of transcription rates for long mRNA *α*_*M*_ and sRNA *α*_*S*_ (S1 Text). Thus, we observed no expression of long mRNA in the regime *α*_*M*_ + *α*_*S*_ < *α*_*A*_, as shown in Figs 3A and 4. We find the threshold to be sharp, and attribute this to the very slow degradation of CsrA compared to long mRNA and sRNA [55, 56].

Apart from the threshold itself, we find that the levels of free CsrA and free sRNA predicted by our steady state analysis are consistent with experimental in-vivo values determined by previous studies [43, 57]. Moreover, our results are also consistent with the total amount of CsrA as well as its ratio to sRNA (S1 Text).

So far, we have demonstrated that our three-component system is capable of producing a threshold behavior. However, it has been shown previously that a mutually exclusive production of sRNA and a target mRNA is possible with just two components [36]. The question thus arises why a third component is needed at all. One possible explanation is that the sRNA makes it easier to trigger lysis, as an increase in sRNA production induces an increase in the abundance of long mRNA (Fig 3).

After SOS signals, the sRNA controls and accelerates the degradation of CsrA (see section on expression dynamics below), eventually leading to the expression of the lysis protein.

### sRNA controls fluctuations close to thresholds

In a next step, we analyzed the stochastic dynamics of the post-transcriptional regulation network. To this end, we switched to a stochastic description, calculated the Fano factor (Var*M*/〈*M*〉) for the abundance of long mRNA (see Materials and Methods), and depicted it as heatmap in Fig 4. The Fano factor measures the relative magnitude of fluctuations, and has already been applied to gene regulatory networks in previous studies [58]. It can also be understood as a quantified comparison with the pure birth process (Poisson process), which has the Fano factor *F* = 1.

We found that fluctuations in mRNA were most pronounced close to the threshold position, with the largest fluctuations occurring slightly above the threshold (Fig 4). Moreover, Fig 4 also shows that the fluctuations became larger as sRNA production decreases. Thus, the third component (sRNA) in the post-transcriptional regulation network also enables significant dampening of fluctuations in long mRNA.

To understand why the fluctuations are localized to the region near threshold, one must take the characteristics of this parameter regime into account. Around the threshold, molecule numbers are close to zero, which has a direct affect on the relative size of fluctuations: the lower the abundance, the larger the fluctuations (stochastic regime). Moreover, the threshold is the only regime in which all three components, CsrA, mRNA and sRNA, can coexist and interact with each other: An increase in the level of CsrA will lead to a decrease in the abundance of long mRNA and sRNA, owing to increased complex formation and subsequent degradation. Analogously, an increase in long mRNA and sRNA molecule numbers leads to a decrease in CsrA abundance. Therefore, the abundance of CsrA dimers is anti-correlated with the abundance of both long mRNA and sRNA. It has been shown for a two-component system, that anti-correlated components can create anomalously large fluctuations [59] if degradation rates are small compared to turnover (ratio of production rate to abundance). For long mRNA, this is exactly the case close to threshold, where the long mRNA abundance is still very low.

These results show that a third component can reduce intrinsic fluctuations of a hierarchically ordered regulatory network.

### Modeling colicin E2 expression dynamics in response to an SOS signal

To study the dynamical response of the ColE2 system to an SOS signal, we extended the post-transcriptional network by including the LexA-RecA regulatory network [53] (Fig 1). LexA not only represses the SOS promoter, it is also an auto-repressor, as well as being a repressor of RecA production. As outlined in the Introduction, RecA forms filaments after DNA damage, which then induce auto-cleavage of LexA dimers. Consequently, the levels of RecA, LexA and the colicin mRNAs increase, as repression due to LexA is relaxed. A stochastic model of this network has been introduced recently [53]. In that study, promoter activity in the LexA-RecA system was found to occur in ordered bursts that result from fluctuations and the particular structure of the RecA-LexA feedback loop.

In our analysis of the ColE2 post-transcriptional regulation network so far (see above), we have assumed the dynamics of SOS promoter activation to be so fast that we could use an effective transcription rate *α*_*M*_ for long mRNA. To link the LexA regulatory network to the post-transcriptional regulation network, we must drop this assumption and explicitly model the dynamics of LexA dimers, which connect the two networks. In the biological system, this involves the binding and dissociation of LexA dimers to and from the SOS promoter in the ColE2 operon. Long mRNA and short mRNA are transcribed only from the derepressed promoter at rates *α*_*M*_*l*__ and *α*_*M*_*s*__, respectively. Thus, the transcription rates of long mRNA and short mRNA are proportional to the number of open SOS promoters in the bacterium. The majority of transcripts are short mRNAs. The mathematical implementation of the integrated regulation network is again a system of coupled rate equations, which we describe in S1 Text. The additional parameters of the LexA-RecA regulation network are to be found in S2 Table.

**Fig 5:**
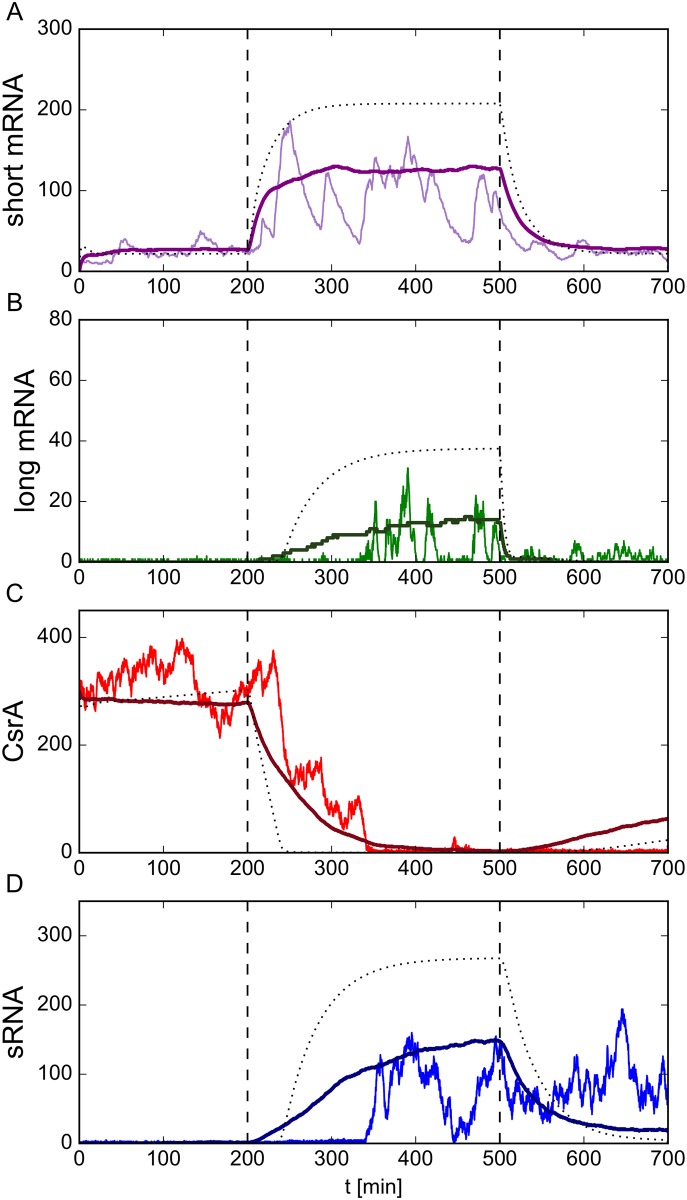
Dynamical behavior before and after a realistic SOS response. We simulated an SOS signal by temporarily up-regulating the LexA auto-cleavage parameter from *c*_*p*_ = 0.0 to *c*_*p*_ = 6.0 between the two dashed vertical lines at *t* = 200 min and *t* = 500 min. The parameter *c*_*p*_ gives the rate at which LexA dimers degrade due to the presence of RecA. During the simulation, we tracked the abundance of (A) free short mRNA, (B) free long mRNA, (C) free CsrA dimers and (D)free sRNA over time. In each panel, the fluctuating colored curve represents a single realization of the stochastic system as implemented by a Gillespie simulation. The smoother darker-colored curve shows the average of 500 different realizations. The black dashed curve depicts the results found by numerical integration of the deterministic rate equations, which neglects fluctuations. In general, the stochastic realizations deviated significantly from both the simulation average and the deterministic solution, as they exhibited large spontaneous bursts. As the short mRNA is not post-transcriptionally regulated, its abundance level can serve as a proxy for the SOS promoter activity. Comparing the free short mRNA abundance with free long mRNA shows that short promoter activity peaks were reliably filtered out by post-transcriptional regulation. After an up-regulation of the LexA auto-cleavage parameter *c*_*p*_ at *t* = 200 min, the abundance of short mRNA rose and is expressed in large bursts. After some time delay, during which all newly produced long mRNAs immediately sequestered CsrA dimers, discrete bursts of free long mRNA are seen, which were followed by periods of no production at all. The timing of the bursts varied considerably between different realizations. A comparison with (C) shows that the abundance of free long mRNA is anti-correlated with the molecule number of all free CsrA. Hence, free long mRNA is only present if the number of free CsrA dimers is low. In the simulation, the production rate of CsrA dimers was set to *α*_*A*_ = 58.52 and the transcription rate of sRNA to *α*_*S*_ = 57.5. All other parameters are given in S1 and S2 Tables.

We simulated the SOS signal by temporarily up-regulating the coupling parameter *c*_*p*_, which quantifies the ability of RecA to induce cleavage of LexA (Fig 1). In the uninduced state before and after the SOS signal, the auto-cleavage parameter was set to *c*_*p*_ = 0. Under SOS stress *c*_*p*_ was increased to *c*_*p*_ = 6. This increase in *c*_*p*_ subsequently boosts the long mRNA production, and therefore relates to a transition from a sub-threshold state (gray area below the white line in Fig 3A) to a super-threshold state (green area above the white line in Fig 3A). Due to the stochasticity in the LexA-RecA network and the resulting stochastic promoter dynamics, the overall transcription rate *α*_*M*_*l*__ of long mRNA is not constant, but fluctuates about a mean value. The production rate of sRNA was held constant at *α*_*S*_ = 57.5. Fig 5 shows the dynamics of short and long mRNA levels and the abundance of CsrA dimers and sRNA in response to transient SOS signaling. When we compared a stochastic realization using Gillespie simulations (Materials and Methods) with a numerical solution of the deterministic rate-equation system, we observed significant qualitative and quantitative differences. First, the stochastic realization exhibited significant fluctuations that manifested themselves in abrupt, short-lived changes in the abundance of short mRNA over the whole time-course (Fig 5A). Second, the average over 500 stochastic realizations deviated from the deterministically predicted value. Both phenomena arise from the intrinsic stochasticity of the LexA-RecA-regulatory network, as explained by Shimoni [53]. Fluctuations may lead to a spontaneous dip in the number of LexA dimers which releases all LexA-regulated genes, including the *lexA* gene itself, from repression. This consequently leads to a sudden rise in the abundance of short mRNA. The open *lexA* and *recA* promoters will then generate a burst of newly produced LexA and RecA proteins, which block and regulate the promoters for the next burst.

Focusing on the dynamics of mRNA transcription, we found that, due to initial simulation parameters, only small numbers of the short mRNA are produced in the uninduced state. After up-regulation of the LexA auto-cleavage parameter *c*_*p*_ at *t* = 200 min, the abundance of short mRNA rises and the aforementioned large bursts appear. The amount of long mRNA, however, follows a completely different trajectory, conditioned by post-transcriptional regulation. Before the SOS signal, expression of long mRNA is almost completely repressed by CsrA (Fig 5B). Even the bursts of SOS promoter activity reflected in fluctuating amounts of the short mRNA have little or no impact on the long mRNA. This filtering effect is biologically relevant, as it ensures that noisy promoter activity does not erroneously trigger lysis. After induction of the SOS signal, the deterministic dynamics of the underlying rate equations predicted that, after a delay of about 40 min, the abundance of long mRNA should rapidly rise to a saturation value (black dashed line in Fig 5B). However, a mean of 500 realizations deviated significantly from this prediction (Fig 5B). In particular, the average number of long mRNA molecules increased more slowly than predicted by deterministic dynamics. Hence the abundance saturated at a much lower value. An appreciable delay between SOS signal induction and expression of long mRNA was still observed, but lasted for only 15 min.

**Fig 6:**
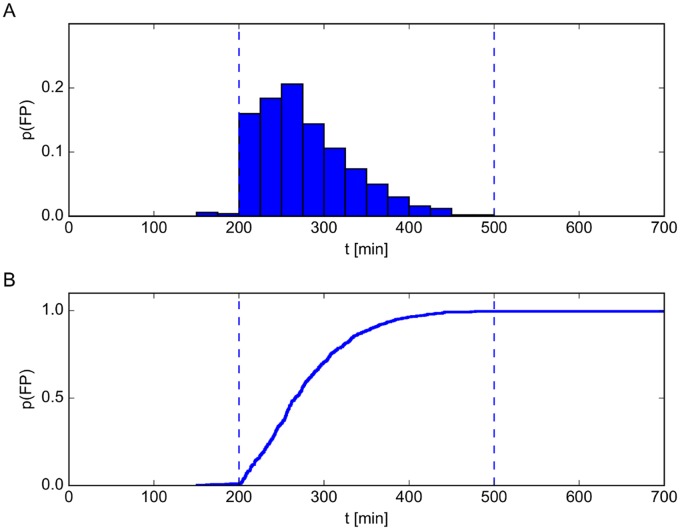
Probability distribution of the first peak in long mRNA abundance and survival function. We simulated an SOS signal by temporarily up-regulating the LexA auto-cleavage parameter from *c*_*p*_ = 0.0 to *c*_*p*_ = 6.0 between the two dashed vertical lines at *t* = 200 min and *t* = 500 min (see also Fig 5). The parameter *c*_*p*_ gives the rate at which LexA dimers degrade due to the presence of RecA. (A) With the parameters defined in S1 and S2 Tables, the timing of the first peak in long mRNA abundance is broadly distributed with maximal probability approximately 60 min after induction of the SOS signal. (B) The survival function is defined as the fraction of *E. coli* cells in a population that exhibited no peak in long mRNA abundance, and thus would not release colicin. The fraction of cells releasing colicin increased smoothly after induction up to 100%. This heterogeneous response of a bacterial population to an SOS signal is also observed in nature. In the simulation, the production rate of CsrA dimers was set to *α*_*A*_ = 58.52 and the transcription rate of sRNA to *α*_*S*_ = 57.5. All other parameters are given in S1 and S2 Tables.

Studying the dynamics of a single stochastic realization, we observed that the number of long mRNA molecules underwent large fluctuations, which were followed by periods of no expression at all. Moreover, the timing of these bursts varied considerably between different realizations. This constitutes a significant qualitative difference compared to the average over 500 realizations and to the deterministic dynamics (Fig 5), both of which exhibit a smooth and continuous temporal behavior. Fig 5B and 5C indicates the origin of this behavior: The abundance of long mRNA can only grow if the number of free CsrA dimers is low. The same holds for the abundance of sRNA, which supports the degradation of CsrA and also can only reach non-zero abundances if there is no CsrA left (Fig 5D). Thus, before any long mRNA can be expressed, the free CsrA concentration must drop to very low values due to degradation or complex formation. The delay between SOS signal induction and the first burst of long mRNA synthesis therefore depends on the amount of CsrA available. We went on to study the precise timing of the first burst in long mRNA abundance, since it is crucial for the time-point of release of colicin-immunity complexes. To this end, we calculated the probability distribution for the first peak from an ensemble of 500 stochastic realizations. The probability of a peak in long mRNA abundance rose quickly and reached its maximum approximately 60 min after induction of the SOS signal (Fig 6A). This phenomenon is also seen in experimental systems: time-lapse studies with colicin-producing bacteria revealed that their lysis time is broadly distributed [48]. The distribution depicted in Fig 6A matches qualitatively with comparable datasets from these experiments. Moreover, our model is able to numerically predict average lysis times in dependence on different SOS signal strengths (see S5 Fig). From the probability distribution of the timing of the initial peak in long mRNA abundance we calculated the survival function, i.e. the probability with respect to time that a cell will not release toxin. Here we assumed that this first burst provides enough long mRNA in the cell to produce the lysis protein, which then induces its lysis with concomitant release of colicin-immunity complexes into the surrounding medium. The function of lysed cells plotted in Fig 6B shows that the number of cells that release the toxin rises with the duration of the SOS signal.

Incorporation of the LexA-RecA regulatory network allowed us to model the colicin E2 expression dynamics in response to a realistic SOS signal, and the results presented above highlight the importance of CsrA for colicin release.

## Discussion

Gene expression is a process that allows for various forms of regulation at all levels. In theoretical studies of post-transcriptional regulation of several biological systems, modulation of mRNA production by proteins or sRNA has been shown to create, for instance, temporal thresholds for mRNA translation [9, 35, 36]. Proteins have also been shown to regulate the expression of the toxin colicin E2 [28] in the context of an SOS response to environmental stress. Experimental studies have elucidated the detailed interaction network responsible for the production and release of the colicin [28]. However, the dynamics of this system, in particular at the post-transcriptional level, have remained elusive. In close analogy to previous two-component models, we developed a mathematical model for this hierarchically ordered post-transcriptional regulation of colicin E2 release. Interestingly, the known interaction network for this system necessitated the modeling of three, not two, components: the long mRNA which is necessary for colicin release, its negative regulator CsrA, and sRNA, which in turn negatively regulates CsrA. Contrary to previous studies [9, 35, 36, 60], the sRNAs do not regulate the mRNA directly, but control the level of the regulator protein CsrA. Thus, the sRNA acts as the “regulator’s regulator”.

In our analysis of the model, we used rate constants that were determined from experimental systems (see chapter 2 of S1 Text for details). Comparing the predicted CsrA levels before the SOS signal (see Fig 5C) with in-vivo measurements of *E. coli* [57] shows that our model results in a pre-SOS free CsrA abundance that agrees with actual bacterial systems (for other abundances, see S1 Text). Moreover, the model is not just able to predict steady state abundances, but also reproduces the reaction to varying external stress levels as seen in experiments (see S5 Fig).

Investigation of the dynamics revealed that the model exhibits a time delay in the production of free long mRNAs. This delay is due to the high abundance of CsrA in the non-SOS state of the cell, which causes CsrA to quickly bind to free long mRNA and thus prevents its transcription. Only during an SOS signal, which indicates external stress for the cell, the level of CsrA gets steadily reduced. The time this process takes to get CsrA levels so low that fluctuations in long mRNA production result in free long mRNA, causes a delay in colicin release. As colicin release is coupled to cell lysis, the delay is therefore a mechanism for filtering out transient SOS signals that might erroneously lead to synthesis of the lysis protein. Moreover, also intrinsic fluctuations, for instance in sRNA production, are filtered out by this mechanism: Even if a large and sudden burst in sRNA were strong enough to drop CsrA abundance close to zero, the CsrA buffer gets restored quickly due to the large production rate of CsrA. This rate is only effectively lowered during a SOS signal, which increases the production of the CsrA-sequestering long mRNA. The fact that lysis is regulated by a threshold mechanism of a global regulator protein like CsrA might also be a guarding mechanism for the cell: only prolongued extreme situations will cause the abundance of these regulators to drop to low molecule numbers.

However, delays and similar threshold behavior also emerge in two-component systems, raising the question why a third component is necessary here. Strikingly, we found that the third component (sRNA) in the post-transcriptional interaction network enables the cell to tune the duration of the delay by sequestering CsrA. In the case of the ColE2 system, this means that cells are able to adjust the (average) time between a SOS signal and the onset of cell lysis leading to colicin release.

Furthermore, previous studies of systems with slow, bursting promoter kinetics have also uncovered a major limitation of two-component sRNA-based regulation compared to regulation based on transcription factors: Two-component systems are subject to significantly higher levels of intrinsic noise [9]. However, Fig 4 (panels A,C,D) shows that, in the post-transcriptional regulation of colicin E2 release, fluctuations become smaller at higher values of *α*_*S*_. The sRNA might therefore allow for significant dampening of these fluctuations. This idea is supported by the fact that the relatively high degradation rate of sRNA makes it less susceptible to induced fluctuations.

In bacteria, these mechanisms could have several functions: First, a comparison of different sRNA production rates (S4 Fig) indicates that the sequestration of CsrA by the sRNA could indeed be crucial for fast release of the colicin, as CsrA degradation rates cannot be arbitrarily increased in bacterial systems. Second, they can tune the reaction to external stress at the population level. Experimental studies have shown that, in the absence of stress, 3% of colicin producing cells release the toxin during the stationary phase; but this fraction can be increased up to eventually 100% if an external SOS stress is applied [14, 48]. Previous experimental studies also found that colicin systems exhibit heterogenous expression times, which originate from the stochasticity of the SOS signal [49, 50]. Recent time-lapse experiments with colicin E2 producing bacteria showed that this lysis time distribution also depends on the strength of the SOS signal [48]. We reproduced these experiments with stochastic simulations, in which we created different stress levels by different values of the RecA degradation rate parameter *c*_*p*_. Our predictions for lysis time distributions (Fig 6A and S5 Fig) show qualitative agreement with these time-lapse experiments. Moreover, the ability of the sRNA to tune the average duration of the delay might serve as a mechanism to adjust the cell lysis to different stress levels. Altering the sRNA level could be an additional mechanism, apart from the stochastic SOS signal, by which bacterial populations can adjust the fraction of cells releasing the toxin depending on the strength and duration of the external stress. Finally, the co-option of sRNA makes the cells less susceptible to lysis due to adventitious fluctuations in promoter activity. This is particularly important considering the bursting behavior and large-scale fluctuations seen in the LexA-RecA-regulatory system, which are readily observed in experiments and reproduced by stochastic models [53].

In order to focus on the interplay between the LexA-RecA system and the hierarchical regulation of long mRNA by CsrA and sRNA, we kept the plasmid number constant. If we considered random, Poisson-distributed plasmid numbers instead, the effect would be very small, as shown in S4B Fig. This fact demonstrates that the colicin plasmid copy number only has minor influence on the lysis time distribution (see S1 Text for details).

In conclusion, we have provided here the first detailed theoretical description of colicin E2 production and release, and used it to study the dynamical behavior of this system. Moreover, the general three-component model described here should be applicable to many other systems of toxin production in microorganisms.

## Materials and Methods

### Derivation of effective long mRNA transcription rate *α*_*M*_

In most models of prokaryotic gene expression, it is assumed that promoter kinetics are fast compared to RNA production and degradation rates. In that case, the promoter state is well approximated by its steady state [54]. In the analysis of the post-transcriptional regulation network, the promoter status affects the transcription rate of the (long) mRNA. Thus, we replaced it by an effective transcription rate for (long) mRNA, which takes into account the probability of a gene being blocked. In the literature this procedure is referred to as “adiabatic elimination of fast variables” (see for example [61]). For this effective rate we also took into account that the colicin operon is located on a plasmid [62], of which approximately 20 copies exist in each cell [14] (see S1 Text).

### Reduction of CsrB and CsrC to an effective sRNA

The two sRNAs CsrB and CsrC regulate CsrA via complex formation. More specifically, each CsrB molecule has approximately 22 binding sites for CsrA, with 9 CsrA dimers being attached on average [63, 64]. CsrC interacts in the same way, but has fewer CsrA binding sites [63]. As a first step, we therefore replaced the two sRNA types by a single effective one, which has N binding sites. However, all of the *N* + 1 sRNA configurations still enter the interaction network as separate components, since the binding and dissociation probabilities change with the number of free binding sites. By investigating the dynamics of the CsrA-sRNA complexes, we discovered that the probability distribution for occupied CsrA binding sites on the sRNAs reaches its stationary state on a time scale that is proportional to the rate of complex-(un)binding. Since binding and unbinding events are biochemically much simpler processes than transcription, translation or degradation, it is very likely that the dynamics of CsrA-sRNA complexes is much faster than all other reaction rates in the system. Following the line of Levine [36] and Legewie [34], we therefore assumed rapid complex dynamics, and replaced the different binding site occupations by an effective sRNA, with only one binding site and transcription rate *Nα*_*S*_ (see S1 Text for details on the calculation).

### Approximate solution of the reduced three-component model

For the calculations of the abundances of the three components (for example, to obtain the plots of Fig 3), we began by assuming the stationary state. Solving for the abundance of one component then gives a cubic equation, for which the exact, general solution is very lengthy and cumbersome to analyze. Therefore, we considered the cubic equation for the cases of very large and very small molecule numbers, and ignored terms that became negligible. This resulted in two easily solvable quadratic equations. Comparisons with numerical solutions of the cubic equations proved that the quadratic solutions approximate the general solution well in their respective abundance regime. Equating the terms omited in the approximation yields a criterion for the transition between the two approximations (see S1 Text). The transition is depicted as a white line in Fig 3. That this transition lies close to the threshold is coincidental. Comparison with the exact, numerical solutions showed that the threshold is not an approximation artifact. S2 Fig illustrates the precision of the approximation by comparing its prediction for long mRNA abundance to that from numerical simulations.

### Calculation of the Fano factor using linear noise approximation

We started the analysis of noise properties by reformulating the simplified three-component system as a Master equation. As Master equations are typically impossible to solve analytically, we performed a general van Kampen expansion in multiple variables (components). Our analysis included all higher orders, and not only lowest order terms as is commonly found in textbooks [61, 65]. With van Kampen’s expansion we were able to derive general formulas for the first up to the fourth moment of the random variable representing the fluctuations of the system around the stationary solution of the rate equations. The terms of each equation were classified in first order terms (dominant terms) and higher order terms (second order, third order, etc), according to the scaling behavior of each term with the system size. We used different methods to calculate the Fano factor for long mRNA. The most reliable results were obtained by implementing only first order terms in the calculations of second moments. This reproduced the shape of the Fano factor well, but it overestimates fluctuations in the vicinity of the threshold. S3 Fig illustrates the degree of agreement between analytical calculations of the Fano factor agree with the results from Gillespie simulations.

### Gillespie simulations

To verify how well our analytical results of the deterministic rate equations coincide with the actual mean molecule numbers, we set up a Gillespie simulation [66]. The Gillespie algorithm generates a statistically correct realization of the master equation behind the rate equations. The core of the algorithm lies in using random numbers to determine which next reaction will occur and the waiting time prior to the succeeding reaction. The reactions simulated by the Gillespie approach are listed in S1 Text. To quantify the delay between SOS signal induction and the first burst in long mRNA abundance, we defined the beginning of the first peak as the point when the number of long mRNA molecules exceeds 8 for the first time. The time of the peak itself was set to the point at which that number reached a maximum. We then calculated the probability distribution from an ensemble of 500 stochastic realizations, using the parameters defined in S1 and S2 Tables.

## Supporting Information

S1 Table Parameter values for the post-transcriptional dynamics modeled by the rate equations and Gillespie simulations. Rates are given in molecules per cell volume *V*_*EC*_ = 0.65*μm*^3^ per minute. The number of ColE2 plasmids is *n*_sos_ = 20. The literature values can be found in [28, 55, 56].
(PDF)

S2 Table Additional parameter values for the SOS response network, modeled by the rate equations and Gillespie simulations. Rates are given in molecules per cell volume *V*_*EC*_ = 0.65*μm*^3^ per minute. The number of ColE2 plasmids is *n*_sos_ = 20. *R*, *Le*,*Col*,*L*: number of RecA proteins, LexA dimers, colicin proteins and lysis proteins. *M*_*l*_,*M*_*r*_,*M*_*s*_,*M*: number of *lexA*, *recA*, short mRNAs and long mRNAs. *B*_*l*_ *B*_*r*_,*B*_sos_: number of LexA dimers bound to the *lexA*, *recA* and SOS promoters. All literature values are taken from [53].
(PDF)

S1 Fig Detailed interaction scheme of post-transcriptional regulation network. The interaction scheme is mathematically formulated as *N* + 5 coupled rate equations. M,A,S,L and C_ma_ give the numbers of long mRNA, CsrA dimers, sRNA, lysis protein and long mRNA-CsrA complexes. C_*n*_ gives the number of sRNA molecules with n CsrA dimers bound. The rates of a reaction is expressed by the formula next to the arrows. *α*: production rates; *δ*: degradation rates; *v*^−^,*k*^−^: Complex dissociation rates; *v*^+^,*k*^+^: Complex formation rates. To illustrate the complex dynamics between CsrA dimers and sRNA we depict the reaction rates of CsrA with an sRNA that has already bound *n* ∈ [0, 1, …, *N*] CsrA dimers. For more details see S1 Text.
(EPS)

S2 Fig Comparison of the stationary solution for long mRNA abundance M* with the time-average 〈*M*〉. The stationary solution M* was calculated using rate equations, the time-average 〈*M*〉 was obtained by Gillespie simulations. We show two cuts through the surface of Fig 3A at *α*_*S*_ = 20 and *α*_*S*_ = 40. The points indicate the result of Gillespie simulations, whereas the lines show the analytical result obtained from the approximated steady state equations. The production rate of CsrA dimers was chosen to be *α*_*A*_ = 58.52, all other parameters are given in Table S1 Table.
(EPS)

S3 Fig Comparison of the analytically calculated Fano factor with corresponding Gillespie simulations. The production rate of CsrA dimers was set to *α*_*A*_ = 58.52. All other parameters are given in S1 Table. For both parameter sets, *α*_*S*_ = 20 and *α*_*S*_ = 40, the analytic calculations using van Kampen’s system size expansion reproduced the shape of the fluctuations obtained by Gillespie simulations well. In the threshold regime the analytic result overestimated the fluctuations slightly.
(EPS)

S4 Fig Effects of parameters on the lysis time distribution. (A) shows the lysis time distribution as in Fig 6A for comparison. (B) This distribution hardly changes if the number of plasmids, *n*_*SOS*_, follows a Poisson distribution. (C) Lowering the sRNA production rate to *α*_*S*_ = 56 shifts the lysis distribution towards later times, whereas (D) doubling it to *α*_*S*_ = 58 causes several cells to lyse even before (and hence independent of) the SOS signal. This illustrates that the sRNA is a possible means of controlling cell lysis.
(EPS)

S5 Fig Average lysis times for different stress levels. To illustrate the predictive possibilities of our three component model, we compare the results of numerical simulations using our model with experimental data [48]. The experiment measured the average lysis time for three different concentrations of the antibiotic Mitomycin C (0.05, 0.25 and 0.70 *μ*g/ml). In the numerical simulations, we used the parameter set defined in S1 and S2 Tables, and varied the parameter *c*_*p*_ (values: 1, 3, 6, 12, 15, 20, 30, 90) to emulate the stress levels. To fit the data, we only applied a scaling factor to map the Mitomycin concentration to values of *c*_*p*_, and shifted the theoretical delays by a constant value. The last step is necessary, as the numerical simulations also account for the constant time between SOS signal and first appearance of short mRNA, which is not the case in the experiments.
(EPS)

S1 Text Supplementary information on calculations and numerical simulations. Detailed derivations of the (simplified) rate equations and the linear noise approximation, as well as the detailed reaction scheme used in the Gillespie simulations.
(PDF)
