# Supplementary material for: Hierarchical Post-transcriptional Regulation of Colicin E2 Expression in Escherichia coli
Source: PLoS Comput Biol. 2016 Dec 15;12(12):e1005243. doi: 10.1371/journal.pcbi.1005243 (PMC5157957; doi:10.1371/journal.pcbi.1005243)
Supplement: S1 Table — Rates are given in molecules per cell volume VEC = 0.65μm3 per minute. The number of ColE2 plasmids is nsos = 20. The literature values can be found in [28, 55, 56]. (PDF) [file pcbi.1005243.s001.pdf]

**Description**	**Rate**	**Parameter**	**Literature**	**Estimates**
Lumped effective transcription and translation rate of CsrA dimers	β_A	β_A	-	58.52
Transcription rate of the effective sRNA	α_S	α_S	-	57.5
Degradation rate of short mRNA	$\delta_{M_s} M_s$	δ_{M_s}	0.04	-
Degradation rate of long mRNA	$\delta_M M$	δ_M	0.04	-
Degradation rate of CsrA dimers	$\delta_A A$	δ_A	0.00007	-
Degradation rate of effective sRNA	$\delta_S S$	δ_S	0.023	-
Coupled degradation rate of long mRNA with CsrA dimers	$k_M MA$	k_M	-	0.5
Coupled degradation rate of effective sRNA with CsrA dimers	$k_S AS$	k_S	-	0.5
Probability of CsrA dimers surviving degradation of sRNA-CsrA complexes	$1 - p_M$	-	0	-
Probability of CsrA dimers surviving degradation of mRNA-CsrA complexes	$1 - p_S$	-	0	-
