# Supplementary material for: Hierarchical Post-transcriptional Regulation of Colicin E2 Expression in Escherichia coli
Source: PLoS Comput Biol. 2016 Dec 15;12(12):e1005243. doi: 10.1371/journal.pcbi.1005243 (PMC5157957; doi:10.1371/journal.pcbi.1005243)
Supplement: S2 Table — Rates are given in molecules per cell volume VEC = 0.65μm3 per minute. The number of ColE2 plasmids is nsos = 20. R, Le,Col,L: number of RecA proteins, LexA dimers, colicin proteins and lysis proteins. Ml,Mr,Ms,M: number of lexA, recA, short mRNAs and long mRNAs. Bl Br,Bsos: number of LexA dimers bound to the lexA, recA and SOS promoters. All literature values are taken from [53]. (PDF) [file pcbi.1005243.s002.pdf]

**Description**	**Rate**	**Parameter**	**Literature**	**Estimates**
Transcription rate of *recA* mRNA	$\alpha_{M_r}(1 - B_r)$	α_{M_r}	3.0	-
Transcription rate of *lexA* mRNA	$\alpha_{M_l}(1 - B_l)$	α_{M_l}	1.8	-
Translation rate of RecA protein	$\beta_R M_r$	β_R	2.4	-
Translation rate of LexA protein	$\beta_{Le} M_l$	β_{Le}	6.0	-
Degradation rate of *recA* mRNA	$\delta_{M_r} M_r$	δ_{M_r}	1.2	-
Degradation rate of *lexA* mRNA	$\delta_{M_l} M_l$	δ_{M_l}	0.18	-
Degradation rate of RecA protein	$\delta_R R$	δ_R	1.2	-
Degradation rate of LexA protein	$\beta_{Le} Le$	β_{Le}	0.12	-
Binding rate of LexA dimers to *recA* promoter sites	$k_r^+(1 - B_r)Le$	k_r^+	1.2	-
Binding rate of LexA dimers to *lexA* promoter sites	$k_l^+(1 - B_l)Le$	k_l^+	0.6	-
Unbinding rate of LexA dimers from *recA* promoter sites	$k_r^- B_r$	k_r^-	2.4	-
Unbinding rate of LexA dimers from *lexA* promoter sites	$k_l^- B_l$	k_l^-	0.6	-
Rate of LexA auto-cleavage due to RecA protein	$c_p R Le$	c_p	-	0-6
Binding rate of LexA dimers to SOS promoter sites	$k_{sos}^+(1 - B_{\text{sos}})Le$	k_{sos}^+	-	1.2
Unbinding rate of LexA dimers from SOS promoter sites	$k_{sos}^- B_{\text{sos}}$	k_{sos}^-	-	1.2
Transcription rate of short mRNA	$\alpha_{M_s}(n_{\text{sos}} - B_{\text{sos}})$	α_{M_s}	-	1.0
Transcription rate of long mRNA	$\alpha_{M_l}(n_{\text{sos}} - B_{\text{sos}})$	α_{M_l}	-	1.0
