# Supplementary material for: Hierarchical Post-transcriptional Regulation of Colicin E2 Expression in Escherichia coli
Source: PLoS Comput Biol. 2016 Dec 15;12(12):e1005243. doi: 10.1371/journal.pcbi.1005243 (PMC5157957; doi:10.1371/journal.pcbi.1005243)
Supplement: S1 Text — Detailed derivations of the (simplified) rate equations and the linear noise approximation, as well as the detailed reaction scheme used in the Gillespie simulations. (PDF) [file pcbi.1005243.s008.pdf]

Supporting Information for: Modelling of Colicin E2 Expression

Mathias Schwarz[1,2], Matthias Lechner[1], Madeleine Opitz[1], Erwin Frey[1]*,

1 Arnold-Sommerfeld-Center for Theoretical Physics and Center for NanoScience, Physics Department, Ludwig-Maximilians-Universität, München, Germany.
2 Institute for Biological and Medical Imaging, Technische Universität München and Helmholtz Zentrum München, Neuherberg, Germany

*** frey@lmu.de**

Contents

1 Rate Equations **4**

1.1 Initial mathematical model 4

1.2 Analysis of sRNA complex dynamics 5

1.2.1 Generating function 6

1.2.2 Solving the differential equation of the generating function . . . 7

1.2.3 Calculating mean and variance 11

1.2.4 Discussion 11

1.3 Simplified mathematical model 12

1.4 Dimensionless form of the rate equations 13

1.5 Stationary solution of free long mRNA abundance 14

1.5.1 Approximation for small and large molecule numbers 15

1.5.2 Combined solution 15

1.6 Threshold properties of long mRNA expression 16

1.6.1 Threshold position 17

1.7 Comparison with Gillespie simulations 17

1.8 Accounting for additional targets of CsrA 18

2 Biological parameter values in post-transcriptional regulation **24**

2.1 Experimental values 24

2.2 Estimations from similar system 25

2.3 Biological estimations 26

3 Gillespie simulations **28**

3.1 Rate equations 29

3.2 Gillespie simulations 29

4 Linear noise approximation **31**

4.1 Definitions 31

4.2 Master equation and rate equation 31

4.3 Kramers-Moyal expansion and van Kampen's expansion 31

4.4 Simplification for post-transcriptional regulation of Colicin release . . . 33

4.5 Calculation of moments 33

4.6 Scaling of terms in the equations of moments 36

4.7 Calculation of the Fano factor of long mRNA 37

Chapter 1

Rate Equations

1.1 Initial mathematical model

The precise interactions in the regulation network of Colicin E2 release were presented in the results part of the main article. Here we show how we derived the simplified rate equations from the detailed regulation network. The following assumptions underly this process:

1. CsrC interacts with CsrA in the same way as CsrB [1]. There are only minor quantitative differences: At 37°C, the half-life of small RNAs CsrB and CsrC are 1.6 min and 4.1 min, respectively [2]. Furthermore, CsrB has more binding sites than CsrC, and it is unknown if the complex-binding kinetics are different for the two sRNAs. However, we assume these differences to be so small that we can describe the qualitative regulation mechanism of the two sRNAs by one effective sRNA. The biological parameters of the effective sRNA are then adapted to the biological parameters of CsrB and CsrC.
2. For the analysis of the post-transcriptional regulation of Colicin E2 release, we will neglect the regulation of transcription and translation concerning long mRNA, CsrA and sRNAs. In most models of prokaryotic gene expression it is assumed that promoter kinetics are fast compared to production and degradation rates, such that the promoter state is well approximated by its steady state [3]. Thus, an effective transcription rate can be introduced that takes into account the probability of a promoter being blocked. The effective rate is smaller than the original rate. In literature this procedure is referred to as adiabatic elimination of fast variables, see for example [4].
3. The system is considered homogeneous, i.e. reaction rates depend only on the total amount of molecule numbers and not on the local concentration of specific molecules.
4. The exact mechanism of CsrA complex degradation is not known. To keep our model as general as possible we will allow for CsrA dimers to survive degradation of the complexes with probability $(1 - p_M)$ in the case of mRNA complexes and with probability $(1 - p_S)$ for sRNA complexes. We choose CsrA to possibly survive complex degradation, since proteins usually have a much longer lifetime.

Notation

- L, M, A: Number of lysis proteins, free long mRNAs and CsrA dimers.
- C_{MA}: Number of long mRNA-CsrA complexes.
- C_n: Number of CsrA-sRNA complexes with n CsrA dimers bound.
- S: Total number of sRNAs (sum over all C_n).
- $\alpha_M, \alpha_A, \alpha_S, \alpha_L$: Effective production of the component denoted by the subscript.
- $\delta_M, \delta_A, \delta_S, \delta_L, \delta_{C_{ma}}$: Degradation rates of the component denoted by the subscript.
- k_M^+, k_M^-, k^+, k^-: Binding rates (+) and unbinding rates (−) of CsrA with mRNAs and sRNAs.

In general, one sRNA has at most N binding sites for CsrA dimers. The total number of sRNAs $S(t)$ is given by the sum over all numbers of complexes $C_n(t)$ with n CsrA dimers bound:

$$S(t) = \sum_{n=0}^{N} C_n(t) \tag{1.1}$$

Rate equations

From the interaction scheme described in the main text we deduce the following rate equations:

$$\dot{L} = \alpha_L M - \delta_L L \tag{1.2}$$

$$\dot{M} = \alpha_M - \delta_M M - k_M{}^+ MA + k_M{}^- C_{MA} \tag{1.3}$$

$$\begin{aligned}\dot{A} &= \alpha_A - \delta_A A - k_M{}^+ MA + k_M{}^- C_{MA} + \delta_{C_{MA}} C_{MA}(1-p_M) \\ &\quad - Ak^+ \sum_{n=0}^{N} C_n(N-n) + k^- \sum_{n=0}^{N} C_n n + \sum_{n=0}^{N} \delta_S C_n n(1-p_S)\end{aligned} \tag{1.4}$$

$$\dot{C_{MA}} = k_M{}^+ MA - k_M{}^- C_{MA} - \delta_{C_{ma}} C_{MA} \tag{1.5}$$

$$\begin{aligned}\dot{C}_n &= \alpha_S \delta_{n,0} + C_{n-1} Ak^+(N-(n-1)) + C_{n+1} k^-(n+1) \\ &\quad - C_n \left[Ak^+(N-n) + k^- n + \delta_S\right]\end{aligned} \tag{1.6}$$

$$C_{-1} = C_{N+1} = 0 \tag{1.7}$$

With the definition of the total number of sRNA molecules in equation (1.1) we find:

$$\dot{S}(t) = \alpha_S - \delta_S S \tag{1.8}$$

1.2 Analysis of sRNA complex dynamics

The rate equations (1.2)-(1.7) give a precise mathematical description. Yet, the coupling of $N+1$ differential equations for sRNA complexes to the dynamics of CsrA makes it hard to analyze the system. In this section, we will calculate the first and second moment of the distribution of occupied binding sites. We will find out that the

time scale at which the stationary distribution is approached is fast compared to production and degradation processes. Consequently, we can simplify the $N+1$ rate equations for the CsrA-sRNA complexes to one effective differential equation. A very helpful tool for this task will be the definition of a generating function.

1.2.1 Generating function

A probability distribution can be characterized by its moments (if they are finite). The moments of the probability distribution of occupied CsrA binding sites $p(n,t) = {C_n(t)}/{S(t)}$ are defined as:

$$\langle n^i \rangle = \sum_{n=0}^{N} \frac{C_n}{S} n^i \qquad \text{with } i = 1, 2, \dots \tag{1.9}$$

A powerful tool to investigate the moments of a probability distribution is to define a probability generating function. In our case, we chose:

$$G(x,t) = \sum_{n=0}^{N} \frac{C_n}{S} x^n \tag{1.10}$$

The useful property of a generating function is that it encodes the information of all $p(n)$ in one variable x. Consequently, the $N+1$ coupled rate equations for sRNA dynamics are simplified to one differential equation of $G(x,t)$ in the variable x. Once we have found the solution of the generating function, we can calculate the mean number and the variance of occupied binding sites via:

$$\langle n(t) \rangle = \partial_x G(x,t)|_{x=1} \tag{1.11}$$

$$\langle n^2(t) \rangle = \partial_x (x \partial_x G(x,t))|_{x=1} \tag{1.12}$$

Furthermore, we can calculate the probability that n CsrA binding sites are occupied:

$$p(n) = \frac{C_n(t)}{S(t)} = \frac{1}{n!} \partial_x^n G(x,t)|_{x=0} \tag{1.13}$$

Our next goal is to set up a differential equation for $G(x,t)$ and to solve this equation. Afterwards, we will infer on the result to obtain information on the probability

distribution $p(n)$. The time evolution for $G(x,t)$ reads as follows:

$$\begin{aligned}
\frac{d}{dt}G(x,t) &= \sum_{n=0}^{N} \frac{d}{dt}\left(\frac{C_n}{S}\right)x^n = \sum_{n=0}^{N}\left(\frac{\dot{C}_n}{S} - \frac{C_n(\alpha_S - \delta_S S)}{S^2}\right)x^n \\
&= \frac{1}{S}\sum_{n=0}^{N}\Big(\alpha_S\delta_{n,0} + C_{n-1}Ak^+(N-(n-1)) + C_{n+1}k^-(n+1) \\
&\qquad - C_n\left[Ak^+(N-n) + k^-n + \delta_S\right] - C_n\frac{\alpha_S}{S} + C_n\delta_S\Big)x^n \\
&\overset{(1.7)}{=} \frac{1}{S}\sum_{n=0}^{N}\Big(\alpha_S\delta_{n,0} + C_nAk^+(N-n)x + C_nk^-n\frac{1}{x} \\
&\qquad - C_n\left[Ak^+(N-n) + k^-n\right] - C_n\frac{\alpha_S}{S}\Big)x^n \\
&= \sum_{n=0}^{N}\left(\frac{\alpha_S}{C_n}\delta_{n,0} + Ak^+(N-n)(x-1) + k^-n\left(\frac{1}{x}-1\right) - \frac{\alpha_S}{S}\right)\frac{C_n}{S}x^n \\
&= \sum_{n=0}^{N}\left(\frac{\alpha_S}{C_n}\delta_{n,0} + Ak^+(x-1)(N - x\partial_x) + k^-(1-x)\partial_x - \frac{\alpha_S}{S}\right)\frac{C_n}{S}x^n \\
\frac{d}{dt}G(x,t) &= \left(Ak^+(x-1)(N-x\partial_x) + k^-(1-x)\partial_x - \frac{\alpha_S}{S(t)}\right)G(x,t) + \frac{\alpha_S}{S(t)} \qquad (1.14)
\end{aligned}$$

The differential equation (1.14) may be solved using the methods of characteristics. To this end we rewrite equation (1.14):

$$\underbrace{\left(-\frac{\alpha_S}{S(t)} + Ak^+(x(t)-1)N\right)G(x(t),t) + \frac{\alpha_S}{S(t)}}_{\frac{d}{dt}G(x(t),t)} =$$
$$\underbrace{(Ak^+x(t) + k^-)(x(t)-1)}_{\frac{dx(t)}{dt}}\partial_x G(x(t),t) + \partial_t G(x(t),t)$$

We end up with two differential equations of the form:

$$\frac{dx(t)}{dt} = (Ak^+x(t) + k^-)(x(t)-1) \qquad (1.15)$$

$$\frac{d}{dt}G(x(t),t) = \left(-\frac{\alpha_S}{S(t)} + Ak^+(x(t)-1)N\right)G(x(t),t) + \frac{\alpha_S}{S(t)} \qquad (1.16)$$

1.2.2 Solving the differential equation of the generating function

Without loss of generality we set $t_0 = 0$.

Differential equation in $x(t)$

The differential equation (1.15) can readily be solved by separation of variables:

$$\int_0^t dt = t = \int_{x_0}^{x} \frac{dx'}{(x'-1)(k^+Ax'+k^-)}$$
$$= \frac{1}{k^-+Ak^+} \log\left(\frac{|x-1|}{k^-+Ak^+x}\frac{k^-+Ak^+x_0}{|x_0-1|}\right)$$
$$x(t) = \frac{k^- + e^{t\left(k^-+Ak^+\right)}\left(x_0-1\right)k^- + Ax_0k^+}{k^- + A\left(x_0 - e^{t(k^-+Ak^+)}\left(x_0-1\right)\right)k^+} \tag{1.17}$$
$$x_0 = \frac{(x-1)k^- + e^{t\left(k^-+Ak^+\right)}\left(k^-+Axk^+\right)}{-A(x-1)k^+ + e^{t(k^-+Ak^+)}\left(k^-+Axk^+\right)} \tag{1.18}$$

Note here that both choices for $|x-1| = x-1$ or $1-x$ and $|x_0-1| = x_0-1$ or $1-x_0$ yield the same result if we use the same sign convention for x and x_0.

Differential equation in $G(x,t)$

Solving equation (1.16) is more tedious, since it contains the time-dependent inhomogeneity $\alpha_S/S(t)$. The general solution of such an inhomogeneous differential equation is given by the sum of the general solution $G_h(x,t)$ of the homogeneous differential equation, which neglects the inhomogeneity $\alpha_S/S(t)$, and a particular solution $G_p(x,t)$ of the inhomogeneous differential equation, which takes into account the inhomogeneity. Thus, we have:

$$G(x,t) = G_h(x,t) + G_p(x,t) \tag{1.19}$$

Homogeneous solution

We start by solving the homogeneous differential equation:

$$\frac{d}{dt}G_h(x(t),t) = \left(-\frac{\alpha_S}{S(t)} + Ak^+(x(t)-1)N\right)G_h(x(t),t) \tag{1.20}$$

We can set as initial condition $G_0 \overset{!}{=} x_0^{n_0}$, meaning that at $t_0 = 0$ there are only complexes with n_0 CsrAs bound to it. Since there is one degree of freedom in the choice of $G_p(x(t),t)$ (we may add $G_h(x,t)$ multiplied by an arbitrary constant), we can choose $G_p(x_0,0) = 0 \rightarrow G_0 = G_h(x_0,0) = G(x_0,0) \overset{!}{=} x_0^{n_0}$. It follows:

$$\int_{G_0}^{G}\frac{dG_h'}{G_h'} = \int_0^t dt'\left(-\frac{\alpha_S}{S(t')} + Ak^+(x(t')-1)N\right)$$
$$G_h(x,t) = e^{-u(t)}(k^-+Ak^+)^N x_0^{n_0}\left(\frac{e^{(k^-+Ak^+)t}(k^-+Ak^+)^2}{Ak^+(1-x)+(k^-+Ak^+x)e^{(k^-+Ak^+)t}}\right)^{-N}$$
$$= e^{-u(t)}\left(k^-+Ak^+\right)^N\left(\frac{(x-1)k^- + e^{t\left(k^-+Ak^+\right)}\left(k^-+Axk^+\right)}{A(1-x)k^+ + e^{t(k^-+Ak^+)}\left(k^-+Axk^+\right)}\right)^{n_0}\cdot$$
$$\cdot\left(\frac{e^{(k^-+Ak^+)t}(k^-+Ak^+)^2}{Ak^+(1-x)+(k^-+Ak^+x)e^{(k^-+Ak^+)t}}\right)^{-N} \tag{1.21}$$

$$u(t) = \int_0^t dt' \frac{\alpha_S}{S(t')} \tag{1.22}$$

For the integration over time we have introduced $x(t)$ explicitly. Following integration we have replaced every x_0 by the right-hand side of equation (1.18). The term $u(t)$ is the integral over the inhomogeneity that we leave untouched for the moment. When we look at the long-time limit, $u(t)$ will simplify significantly.

To simplify equation (1.21), we set $n_0 = 0$. This choice will not limit our analysis, because we are only interested in the stationary binding site distribution and in the time scale at which the stationary binding site distribution is approached. Both objects of interest are independent of n_0. Hence, the general homogeneous solution is given by:

$$G_h(x,t) = e^{-u(t)} \left(\frac{e^{t\left(k^- + Ak^+\right)} \left(k^- + Ak^+\right)}{-A(-1+x)k^+ + e^{t\left(k^- + Ak^+\right)} \left(k^- + Axk^+\right)} \right)^{-N} \tag{1.23}$$

$$G_h(x_0,t) = e^{-u(t)} \left(\frac{k^- + Ak^+}{k^- + A\left(-e^{t\left(k^- + Ak^+\right)} (x_0 - 1) + x_0\right) k^+} \right)^{N} \tag{1.24}$$

Particular solution

As commonly done, we choose the ansatz $G_p(x(t),t) = G_{pp}(t) G_h(t)$ for the particular solution of equation (1.16). This ansatz leads to a differential equation for the time-dependent parameter $G_{pp}(t)$:

$$\frac{d}{dt} G_{pp}(x(t),t) = \frac{\alpha_S}{S(t) G_h(x(t),t)} \tag{1.25}$$

Thus, we find:

$$G_{pp}(x(t),t) = \int_0^t dt' \underbrace{\frac{\alpha_S}{S(t')} e^{u(t')}}_{\frac{d}{dt'} e^{u(t')}} \left(\frac{e^{t\left(k^- + Ak^+\right)} \left(k^- + Ak^+\right)}{A(x(t)-1)k^+ + e^{t\left(k^- + Ak^+\right)} \left(k^- + Ax(t)k^+\right)} \right)^{N}$$

$$\begin{aligned}
G_{pp}(x_0,t) &= \int_0^t dt' \left[\frac{d}{dt'} \left(e^{u(t')} \right) \right] \left(\frac{k^- + A\left(-e^{t\left(k^- + Ak^+\right)} (x_0 - 1) + x_0\right) k^+}{k^- + Ak^+} \right)^{N} \\
&\overset{\text{PI}}{=} \left[e^{u(t')} \left(\frac{k^- + Ak^+ x_0 + Ak^+(1-x_0) e^{(k^- + Ak^+)t'}}{k^- + Ak^+} \right)^{N} \right]_0^t \\
&\quad - \int_0^t dt' e^{u(t')} \frac{d}{dt'} \left(\frac{k^- + Ak^+ x_0 + Ak^+(1-x_0) e^{(k^- + Ak^+)t'}}{k^- + Ak^+} \right)^{N} \\
&= \sum_{n=0}^{N} \binom{N}{n} \left(\frac{k^- + Ak^+ x_0}{k^- + Ak^+} \right)^{N-n} \left(\frac{Ak^+(1-x_0)}{k^- + Ak^+} \right)^{n} \\
&\quad \left(\left[e^{u(t')} e^{(k^- + Ak^+)nt'} \right]_0^t - \int_0^t dt' e^{u(t')} (k^- + Ak^+) n e^{(k^- + Ak^+)nt'} \right)
\end{aligned} \tag{1.26}$$

In order to proceed with the calculations we have to specify $u(t)$. Since $\lim_{t\to\infty} S(t) = S_\infty = \frac{\alpha_S}{\delta_S}$, we may calculate $u(t)$ as follows:

$$u(t) = \int_0^t dt' \frac{\alpha_S}{S(t)} = \int_0^t dt' \left(\frac{\alpha_S}{S_\infty} + \delta_u(t)\right) = \delta_S t + \int_0^t dt' \delta_u(t) \tag{1.27}$$

Since $S(t)$ converges exponentially fast towards S_∞, we know that $\delta_u(t)$ has to go exponentially fast to zero as well and we end up with a finite integral $\int_0^\infty dt' \delta_u(t) = \Delta_u$. Thus, for times larger than the time scale given by the degradation of sRNA, we may approximate:

$$u(t) \approx \delta_S t + \Delta_u \tag{1.28}$$

It follows that for large times larger than $1/\delta_S$:

$$\begin{aligned} G_{pp}(x_0,t) = &\sum_{n=0}^{N} \binom{N}{n} \left(\frac{k^- + Ak^+ x_0}{k^- + Ak^+}\right)^{N-n} \left(\frac{Ak^+(1-x_0)}{k^- + Ak^+}\right)^n \\ &\left(\left[e^{u(t)} e^{(k^- + Ak^+)nt} - 1\right] - \left[\frac{(k^- + Ak^+)n}{(k^- + Ak^+)n + \delta_S} e^{\delta_S t' + \Delta_u} e^{(k^- + Ak^+)nt'}\right]_0^t\right) \\ = &\sum_{n=0}^{N} \binom{N}{n} \left(\frac{k^- + Ak^+ x_0}{k^- + Ak^+}\right)^{N-n} \left(\frac{Ak^+(1-x_0)}{k^- + Ak^+}\right)^n \\ &\left(1 - \frac{(k^- + Ak^+)n}{(k^- + Ak^+)n + \delta_S}\right) \left(e^{u(t)} e^{(k^- + Ak^+)nt} - 1\right) \end{aligned} \tag{1.29}$$

$$\begin{aligned} G_{pp}(x,t) = &\sum_{n=0}^{N} \binom{N}{n} \left(\frac{(k^- + Ak^+ x)e^{(k^- + Ak^+)t}}{(k^- + Ak^+ x)e^{(k^- + Ak^+)t} - Ak^+(x-1)}\right)^{N-n} \\ &\left(\frac{Ak^+(1-x)}{(k^- + Ak^+ x)e^{(k^- + Ak^+)t} - Ak^+(x-1)}\right)^n \\ &\left(\frac{\delta_S}{(k^- + Ak^+)n + \delta_S}\right) \left(e^{u(t)} e^{(k^- + Ak^+)nt} - 1\right) \end{aligned} \tag{1.30}$$

General solution for $n_0 = 0$

Taking everything together we have:

$$G(x,t) = G_h(x,t)(1 + G_{pp}(x,t)) \tag{1.31}$$

$$G_h(x,t) = e^{-u(t)} \left(\frac{(k^- + Ak^+ x)e^{(k^- + Ak^+)t} - Ak^+(x-1)}{e^{(k^- + Ak^+)t}(k^- + Ak^+)}\right)^N \tag{1.32}$$

$$\begin{aligned} G_{pp}(x,t) = &\sum_{n=0}^{N} \binom{N}{n} \left(\frac{(k^- + Ak^+ x)e^{(k^- + Ak^+)t}}{(k^- + Ak^+ x)e^{(k^- + Ak^+)t} - Ak^+(x-1)}\right)^{N-n} \\ &\left(\frac{Ak^+(1-x)}{(k^- + Ak^+ x)e^{(k^- + Ak^+)t} - Ak^+(x-1)}\right)^n \\ &\left(\frac{\delta_S}{(k^- + Ak^+)n + \delta_S}\right) \left(e^{u(t)} e^{(k^- + Ak^+)nt} - 1\right) \end{aligned} \tag{1.33}$$

1.2.3 Calculating mean and variance

With the general solution of the generating function at hand, we can evaluate the mean and the variance of n occupied binding sites:

$$\langle n(t)\rangle = \partial_x G(x,t)|_{x=1} = \left[(1+G_{pp}(x,t))\partial_x G_h(x,t) + G_h(x,t)\partial_x G_{pp}(x,t)\right]_{x=1} \tag{1.34}$$

$$\begin{aligned}\langle (n(t))^2\rangle = \partial_x(x\partial_x G(x,t))|_{x=1} = \partial_x G(x,t)|_{x=1} + x\left[(1+G_{pp}(x,t))\partial_x^2 G_h(x,t)+\right.\\ \left.+2\partial_x G_h(x,t)\partial_x G_{pp}(x,t) + G_h(x,t)\partial_x^2 G_{pp}(x,t)\right]_{x=1}\end{aligned} \tag{1.35}$$

Inserting equations (1.31)-(1.33) into equation (1.34) and equation (1.35) yields:

$$\langle n(t)\rangle = \frac{ANk^+}{\delta_s + k^- + Ak^+}\left(1 - e^{-(k^- + Ak^+)t - u(t)}\right) \tag{1.36}$$

$$\begin{aligned}\langle n_\infty\rangle &= \frac{ANk^+}{\delta_s + k^- + Ak^+}\\ &= \frac{ANk^+}{k^- + Ak^+}\left[1 - \frac{\delta_s}{k^- + Ak^+} + \mathcal{O}\left(\left(\frac{\delta_s}{k^- + Ak^+}\right)^2\right)\right]\end{aligned} \tag{1.37}$$

$$\begin{aligned}\langle n_\infty^2\rangle &= \frac{ANk^+\left(\delta_s + 2\left(k^- + ANk^+\right)\right)}{\left(\delta_S + k^- + Ak^+\right)\left(\delta_s + 2\left(k^- + Ak^+\right)\right)}\\ &= \frac{ANk^+\left(k^- + ANk^+\right)}{\left(k^- + Ak^+\right)^2} - \frac{\left(ANk^+\left(2k^- + A(-1+3N)k^+\right)\right)\delta_s}{2\left(k^- + Ak^+\right)^3}\\ &\quad + \mathcal{O}\left(\left(\frac{\delta_s}{(k^- + Ak^+)^2}\right)^2\right)\end{aligned} \tag{1.38}$$

1.2.4 Discussion

From equation (1.36) we obtain an important result. Again taking $u(t) \approx \delta_S t + \Delta_u$, we find that the mean number of occupied binding sites relaxes with the rate $\delta_S + k^- + Ak^+$ to its stationary value, which is faster than the relaxation of sRNA abundance, happening with rate δ_S. The degradation of sRNA happens on a time scale of several minutes, whereas binding and unbinding of molecules occurs within several seconds. Thus, $\delta_S \ll k^- + Ak^+$ most likely holds, which has important consequences: *The relaxation of occupied binding sites towards its equilibrium value is so fast that it can be considered in quasi-equilibrium compared to production and degradation processes. Consequently, the dynamics of sRNA complexes may be approximated by its equilibrium distribution.*

If $\delta_S \ll k^- + Ak^+$, the 0th order term in $\delta_s/(k^- + Ak^+)$ in equation (1.37) dominates and we end up with the results for a simple random walk on N sites with hopping probability $p = \frac{Ak^+}{k^- + Ak^+}$ to the right and hopping probability $q = \frac{k^-}{k^- + Ak^+}$ to the left. For such a process, we have: $\langle n\rangle = Np$ and $\mathrm{Var}[n] = \langle n^2\rangle - \langle n\rangle^2 = Npq$, which is reproduced by the 0th order term in equation (1.38).

The mean number of occupied binding sites decreases with increasing δ_S. This makes sense, since an sRNA is always produced with no CsrA dimer bound, i.e. the source of sRNAs pulls the mean towards lower values.
The faster sRNAs degrade, the less molecules are able to bind multiple CsrA molecules before they degrade.

1.3 Simplified mathematical model

With the results from above we can significantly simplify the rate equations (1.2)-(1.7). First, we note that the rate equation for lysis proteins (1.2) is a linear differential equation that depends only on the number of long mRNA molecules, the translation rate α_L and the degradation rate δ_L. Thus, once we understand the dynamics of M, we comprehend the dynamics of L as well. That is why we ignore equation (1.2), leaving us with the following set of equations:

$$\dot{M} = \alpha_M - \delta_M M - {k_M}^+ MA + {k_M}^- C_{MA} \tag{1.39}$$

$$\begin{aligned} \dot{A} &= \alpha_A - \delta_A A - {k_M}^+ MA + {k_M}^- C_{MA} + \delta_{C_{MA}} C_{MA}(1 - p_M) \\ &\quad - Ak^+ \sum_{n=0}^{N} C_n (N-n) + k^- \sum_{n=0}^{N} C_n n + \sum_{n=0}^{N} \delta_S C_n n (1 - p_S) \end{aligned} \tag{1.40}$$

$$\dot{C_{MA}} = {k_M}^+ MA - {k_M}^- C_{MA} - \delta_{C_{ma}} C_{MA} \tag{1.41}$$

$$\begin{aligned} \dot{C}_n &= \alpha_S \delta_{n,0} + C_{n-1} Ak^+ (N - (n-1)) + C_{n+1} k^- (n+1) \\ &\quad - C_n \left[Ak^+ (N-n) + k^- n + \delta_S \right] \end{aligned} \tag{1.42}$$

$$\dot{S} = \alpha_S - \delta_S S \tag{1.43}$$

In section 1.2.4 we found that the probability distribution of occupied CsrA binding sites on sRNA is approached on the time scale $\delta_S + k^- + Ak^+$. As in the work of Levine [5] and Legewie [6] we assume now rapid equilibrium of complex dynamics and approximate the quasi-equilibrated CsrA binding sites distribution by a single, effective complex configuration that has exactly $\langle n_\infty \rangle$ molecules bound:

$$C_0, C_1, \ldots, C_N \to C_{\langle n_\infty \rangle} \qquad \text{with } \langle n_\infty \rangle = \frac{ANk^+}{\delta_S + k^- + Ak^+} \tag{1.44}$$

As all sRNAs are assumed to have this distribution, it holds

$$C_{\langle n_\infty \rangle} \equiv S \tag{1.45}$$

The number of CsrA-mRNA complexes relaxes as well on a time scale proportional to the complex binding and unbinding rates $A{k_M}^+$ and ${k_M}^-$. Consequently, we set the left-hand side of equation (1.41) equal to zero:

$$\dot{C_{MA}} = 0 : \quad C_{MA} = \frac{{k_M}^+ MA}{{k_M}^- + \delta_{C_{MA}}} = \frac{k_M MA}{\delta_{C_{MA}}} \tag{1.46}$$

For a clear notation, we defined the lumped complex dynamic parameters:

$$k_M = \frac{{k_M}^+ \delta_{C_{MA}}}{{k_M}^- + \delta_{C_{MA}}} \tag{1.47}$$

$$k = \frac{k^+ \delta_S}{k^- + \delta_S} \tag{1.48}$$

These lumped parameters can be understood as the effectiveness of coupled degradation, for it is the ratio of binding rate times degradation rate divided by the unbinding rate of the complex. k will be used later on. Taking everything together, we find:

$$\dot{M} = \alpha_M - \delta_M M - k_M MA \tag{1.49}$$

$$\dot{A} = \alpha_A - \delta_A A - p_M k_M MA - p_S \frac{\delta_S k^+}{\delta_S + k^- + Ak^+} N C_{\langle n_\infty \rangle} A \tag{1.50}$$

$$\dot{S} \equiv \dot{C}_{\langle n_\infty \rangle} = \alpha_S - \delta_S C_{\langle n_\infty \rangle} \tag{1.51}$$

Since we switched to a description in which all sRNAs have the same effective binding site occupation, we can perform another simplifying step: Instead of considering $S \equiv C_{\langle n_\infty \rangle}$ sRNAs with N binding sites each, we change to $NC_{\langle n_\infty \rangle}$ sRNAs with a single binding site. In other words, we consider each binding site a separate particle. Then, the total number of sRNAs can then be written as sum of free (unbound) sRNAs (S_{free}) and sRNA-CsrA complexes(C_{AS}).

$$NC_{\langle n_\infty \rangle} = S_{\text{free}} + C_{AS}. \tag{1.52}$$

By "adding a zero", we can write the time derivative of $N\dot{C}_{\langle n_\infty \rangle}$ as:

$$N\dot{C}_{\langle n_\infty \rangle} = \dot{S}_{\text{free}} + \dot{C}_{AS} \tag{1.53}$$

$$\dot{S}_{\text{free}} = C_{AS}k^- - S_{\text{free}}Ak^+ + N\alpha_S - \delta_S S_{\text{free}} \tag{1.54}$$

$$\dot{C}_{AS} = -C_{AS}k^- + S_{\text{free}}Ak^+ - \delta_S C_{AS} \tag{1.55}$$

Assuming fast complex dynamics, we find:

$$\dot{C}_{AS} = 0 \longrightarrow C_{AS} = \frac{S_{\text{free}}Ak^+}{k^- + \delta_S} \tag{1.56}$$

$$\dot{S}_{\text{free}} = N\alpha_S - kAS_{\text{free}} - \delta_S S_{\text{free}} \tag{1.57}$$

Then it follows:

$$\frac{\delta_S k^+}{\delta_S + k^- + Ak^+} NC_{\langle n_\infty \rangle} = \frac{\delta_S k^+}{\delta_S + k^- + Ak^+}(S_{\text{free}} + C_{AS}) \tag{1.58}$$

$$= \frac{\delta_S k^+}{\delta_S + k^- + Ak^+} S_{\text{free}} \frac{k^- + \delta_S + Ak^+}{k^- + \delta_S} \tag{1.59}$$

$$= \frac{k^+ \delta_S}{k^- + \delta_S} S_{\text{free}} = kS_{\text{free}} \tag{1.60}$$

To obtain a clear and concise notation, we redefine $S_{\text{free}} \to S$ and $k \to k_S$, which leads to the very simple rate equations:

$$\boxed{\begin{aligned} \dot{M} &= \alpha_M - \delta_M M - k_M MA && (1.61)\\ \dot{A} &= \alpha_A - \delta_A A - k_M p_M MA - k_S p_S AS && (1.62)\\ \dot{S} &= N\alpha_S - \delta_S S - Ak_S S && (1.63) \end{aligned}}$$

1.4 Dimensionless form of the rate equations

It proved beneficial to work with a dimensionless form of the rate equations (3.9)-(3.11). We start by introducing characteristic time and molecule numbers $t = \tau t_c,\ M = mm_c,\ A = aa_c,\ S = ss_c$ and find:

$$m' = \alpha_M \frac{t_c}{m_c} - \delta_M t_c \cdot m - k_M t_c a_c \cdot ma \tag{1.64}$$

$$a' = \alpha_A \frac{t_c}{a_c} - \delta_A t_c \cdot a - p_M k_M t_c m_c \cdot ma - p_S k_S t_c s_c \cdot as \tag{1.65}$$

$$s' = \alpha_S \frac{t_c}{s_c} - \delta_S t_c \cdot s - k_S t_c a_c \cdot as \tag{1.66}$$

Building on these equations, we have various possibilities to proceed. There are 10 parameters, which we could reduce to 6 parameter combinations. Yet, we would still like to count molecule numbers in the same units, i.e. $m_c = a_c = s_c$. Hence, the number of lumped parameters will decrease by two to a final number of 8.

As a next step, we reduce the number of free parameters even further by dividing the differential equations by suitable parameters. This is also an opportunity to introduce small ratios that can be used for an expansion later on. In particular, we take advantage of the fact that sRNAs, mRNAs and proteins in the regulation network have quite different degradation rates. We choose $t_c = \frac{1}{\delta_M}$, which will lead to a small ratio $\delta_{am} := \frac{\delta_A}{\delta_M}$. Later on, we will use this ratio as an expansion parameter.

Furthermore we would like to simplify the interaction terms and choose $m_c = a_c = s_c = \frac{\delta_M}{k_M}$, which results in:

$$m' = \frac{\alpha_M k_M}{{\delta_M}^2} - m - ma \tag{1.67}$$

$$a' = \frac{\alpha_A k_M}{{\delta_M}^2} - \frac{\delta_A}{\delta_M} a - p_M am - p_S \frac{k_S}{k_M} as \tag{1.68}$$

$$s' = \frac{\alpha_S k_S}{{\delta_M}^2} - \frac{\delta_S}{\delta_M} s - \frac{k_S}{k_M} as \tag{1.69}$$

To further simplify we define $\alpha_m := \frac{\alpha_M k_M}{{\delta_M}^2}$, $\alpha_a := \frac{\alpha_A k_M}{{\delta_M}^2}, \alpha_s := \frac{\alpha_M k_M}{{\delta_M}^2}$ and $k_{sm} = \frac{k_S}{k_M}$. Thus, we find

$$\boxed{\begin{aligned} m' &= \alpha_m - m - ma && (1.70)\\ a' &= \alpha_a - \delta_{am} a - p_M am - p_S k_{sm} as && (1.71)\\ s' &= \alpha_s - \delta_{sm} s - k_{sm} as && (1.72)\end{aligned}}$$

The coupled equations (1.70)-(1.72) are easier to analyze, compared to the original rate equations, but we have to be aware of the dependencies of our newly defined parameters α_m, α_a and α_s on k_M and δ_M.

1.5 Stationary solution of free long mRNA abundance

We start with equations (1.71) and (1.72) and solve the resulting quadratic equation. We discard the solution with negative molecule numbers and find:

$$\begin{aligned} a^* = \frac{1}{2k_{sm}(\delta_{am} + m^* p_M)} \Big[&- \alpha_s k_{sm} p_S + \alpha_a k_{sm} - \delta_{am}\delta_{sm} - \delta_{sm} m^* p_M + \\ &+ \sqrt{(\alpha_s k_{sm} p_S - \alpha_a k_{sm} + \delta_{am}\delta_{sm} + \delta_{sm} m^* p_M)^2 + 4\alpha_a \delta_{sm} k_{sm}(\delta_{am} + m^* p_M)}\Big] \end{aligned} \tag{1.73}$$

$$\begin{aligned} s^* = \frac{1}{2\delta_{sm} k_{sm} p_S} \Big[&\alpha_s k_{sm} p_S - \alpha_a k_{sm} - \delta_{am}\delta_{sm} - \delta_{sm} m^* p_M + \\ &+ \sqrt{(\alpha_s k_{sm} p_S - \alpha_a k_{sm} + \delta_{am}\delta_{sm} + \delta_{sm} m^* p_M)^2 + 4\alpha_a \delta_{sm} k_{sm}(\delta_{am} + m^* p_M)}\Big] \end{aligned} \tag{1.74}$$

Inserting equation (1.73) into equation (1.70) leaves us with a radical equation for m^*. Isolating the square root and taking the square on both sides yields:

$$\begin{aligned}0 = k_{sm}(\delta_{am} + m^* p_M)\big[&\alpha_s k_{sm} m^* p_S(\alpha_m - m^*) + \\ &(\alpha_m k_{sm} + m^*(\delta_{sm} - k_{sm}))(\alpha_m(\delta_{am} + m^* p_M) - m^*(\alpha_a + \delta_{am} + m^* p_M))\big]\end{aligned} \tag{1.75}$$

Since $\delta_{am} > 0$ and $m^* \geq 0$, we can discard the solution $m^* = -\delta_{am}$ as unphysical and divide by this solution. We now expand the equation and sort the terms in the order of m^*:

$$\begin{aligned}0 = &(m^*)^3 p_M(k_{sm} - \delta_{sm}) + \\ &(m^*)^2[\delta_{sm}(\alpha_m p_M - \alpha_a - \delta_{am}) + k_{sm}(\alpha_a - 2\alpha_m p_M - \alpha_s p_S + \delta_{am})] + \\ &(m^*)^1 \alpha_m[k_{sm}(\alpha_m p_M + \alpha_s p_S - \alpha_a) + \delta_{am}(\delta_{sm} - 2k_{sm})] + \\ &(m^*)^0 \alpha_m^2 \delta_{am} k_{sm} \\ =: &(m^*)^3 \mathcal{M}_3 + (m^*)^2 \mathcal{M}_2 + (m^*)^1 \mathcal{M}_1 + \mathcal{M}_0\end{aligned} \tag{1.76}$$

The solutions of this cubic equation can be calculated exactly. Solutions that do not satisfy the original radical solution then have to be discarded. However, the general solution of a cubic equation is very lengthy, and its explicit form does not reveal much of physics or lead to a deeper understanding. That is why we would like to find an easier, approximate solution that allows us to analyze the analytic findings.

1.5.1 Approximation for small and large molecule numbers

For small molecule numbers of long mRNA we can neglect the cubic term in equation (1.76). This leaves us with a quadratic equation whose solution is given by:

$$m^*_{\ll} = \frac{1}{-2\mathcal{M}_2}\left(\mathcal{M}_1 + \sqrt{\mathcal{M}_1^2 - 4\mathcal{M}_2\mathcal{M}_0}\right) \tag{1.77}$$

For large m^*, we neglect the term $(m*)^0 = 1$ in equation (1.76):

$$m^*_{\gg} = \frac{1}{2\mathcal{M}_3}\left(-\mathcal{M}_2 - \sqrt{\mathcal{M}_2^2 - 4\mathcal{M}_3\mathcal{M}_1}\right) \tag{1.78}$$

1.5.2 Combined solution

Having found two solutions for the two regimes $m^* \ll 1$ and $m^* \gg 1$, the question arises how these solutions may be combined to form one function that describes the stationary solution of m^* over the whole parameter range. In the two limiting cases, we neglected for small molecule numbers the cubic term $\mathcal{M}_3(m^*)^3$, and for larger molecule numbers the term $\mathcal{M}_0$. When $\mathcal{M}_3(m^*_{\text{trans}})^3 = \mathcal{M}_0$, these two terms have exactly the same magnitude and, as a consequence, yield exactly the same result for m^*. We use this fact to introduce a transition from $m^*_{\ll}$ to $m^*_{\gg}$ and define:

$$m^* := \begin{cases} m^*_{\ll} & ; m^*_{\ll} < m^*_{\text{trans}} \\ m^*_{\gg} & ; \text{else} \end{cases} \quad \text{for } \delta_{sm} < k_{sm} \tag{1.79}$$

$$m^*_{\text{trans}} = \left(\frac{\mathcal{M}_0}{\mathcal{M}_3}\right)^{1/3} = \left(\frac{\alpha_m^2 \delta_{am} k_{sm}}{p_M(k_{sm} - \delta_{sm})}\right)^{1/3} \tag{1.80}$$

With the solution of m^* and equations (1.73) and (1.74), we obtain a^* and s^*.

There is a minor setback, since in the regime $\delta_{sm} > k_{sm}$ the transition molecule number m^*_{trans} becomes negative. That is why equation (1.79) is only defined for $\delta_{sm} < k_{sm}$.

In the regime $\delta_{sm} > k_{sm}$ we have to follow a different approach. So far, we derived a cubic equation for m^*, but we can also do the same for s^*. Following the same steps as above, while interchanging the roles of m and s, gives rise to another cubic equation:

$$\begin{aligned}
0 = {} & (s^*)^3 \delta_{sm} k_{sm} p_S (\delta_{sm} - k_{sm}) + \\
& (s^*)^2 (k_{sm}(-\alpha_m \delta_{sm} p_M + \alpha_s p_S (k_{sm} - 2\delta_{sm}) + \alpha_a(\delta_{sm} - k_{sm})) + \delta_{am}\delta_{sm}(\delta_{sm} - k_{sm})) + \\
& (s^*)^1 \alpha_s (k_{sm}(\alpha_m p_M + \alpha_s p_S - \alpha_a) + \delta_{am}(k_{sm} - 2\delta_{sm})) + \\
& (s^*)^0 \alpha_s^2 \delta_{am} \\
=: {} & (s^*)^3 \mathcal{S}_3 + (s^*)^2 \mathcal{S}_2 + (s^*)^1 \mathcal{S}_1 + \mathcal{S}_0
\end{aligned} \tag{1.81}$$

In the same way as for the cubic equation in m^*, we can now calculate two limiting solutions for $s^* \ll 1$ and $s^* \gg 1$ that we call $s^*_{\ll}$ and $s^*_{\gg}$:

$$s^*_{\ll} = \frac{1}{-2\mathcal{S}_2} \left(\mathcal{S}_1 + \sqrt{\mathcal{S}_1^2 - 4\mathcal{S}_2\mathcal{S}_0} \right) \tag{1.82}$$

$$s^*_{\gg} = \frac{1}{-2\mathcal{S}_3} \left(\mathcal{S}_2 + \sqrt{\mathcal{S}_2^2 - 4\mathcal{S}_3\mathcal{S}_1} \right) \tag{1.83}$$

The transition between these solutions takes place at:

$$s^*_{\text{trans}} = \left(\frac{\alpha_s^2 \delta_{am}}{\delta_{sm} k_{sm} p_S (\delta_{sm} - k_{sm})} \right)^{1/3} \tag{1.84}$$

which is positive for $\delta_{sm} > k_{sm}$, as opposed to m^*_{trans}. We further define:

$$s^* := \begin{cases} s^*_{\ll} & ; s^*_{\ll} < s^*_{\text{trans}} \\ s^*_{\gg} & ; \text{else} \end{cases} \qquad \text{for } \delta_{sm} > k_{sm} \tag{1.85}$$

From s^* we may than calculate a^*, and finally m^*. Thus, we are able to find an approximate analytic solution for the whole range of parameters ($\delta_{sm} \gtrless k_{sm}$). Comparison with the numerical solution of the cubic equation shows that the approximation is very exact.

The advantage of our approximate solution compared to the exact solution of the cubic equation (1.76) is twofold. First, due to its simple form we are able to understand the equation and are in the position to predict, for example, the dependence of the threshold on specific parameters (see section 1.6). Second, when studying fluctuations we have to deal with long equations that contain the stationary solutions m^*, a^*, s^* as parameters. The simpler the stationary solutions, the quicker are the calculations.

1.6 Threshold properties of long mRNA expression

In the main text we found a distinct threshold in the expression of long mRNA. In this section we will study the threshold properties of the stationary solution m^* in great detail. The analysis will be based on equation (1.79) in general and equation (1.77) in

particular. Equation (1.77) describes well the stationary solution below threshold, from which we can deduce threshold properties. We already defined:

$$\mathcal{M}_3 = p_M(k_{sm} - \delta_{sm}) \tag{1.86}$$
$$\mathcal{M}_2 = [\delta_{sm}(\alpha_m p_M - \alpha_a - \delta_{am}) + k_{sm}(\alpha_a - 2\alpha_m p_M - \alpha_s p_S + \delta_{am})] \tag{1.87}$$
$$\mathcal{M}_1 = \alpha_m[k_{sm}(\alpha_m p_M + \alpha_s p_S - \alpha_a) + \delta_{am}(\delta_{sm} - 2k_{sm})] \tag{1.88}$$
$$\mathcal{M}_0 = \alpha_m^2 \delta_{am} k_{sm} \tag{1.89}$$

The solution given by (1.79) is only valid in the regime $\delta_{sm} < k_{sm}$. In the regime $\delta_{sm} > k_{sm}$, we would have to work with the approximate solution of s^* (equation (1.85)), which complicates the threshold analysis in m^*. However, there is no obvious reason why the dependence of threshold properties should be different in the two parameter ranges $\delta_{sm} < k_{sm}$ and $\delta_{sm} > k_{sm}$.

1.6.1 Threshold position

For small α_M and α_S, the terms $\mathcal{M}_1$ and $\mathcal{M}_2$ are negative. The negative $\mathcal{M}_1$-term in front of the square root is compensated by the same term squared under the root. Neglecting the second term under the square root, this would result in $m^* = 0$ for $\mathcal{M}_1 < 0$ and linear increase of m^* once $\mathcal{M}_1$ becomes positive. This is how the threshold is encoded in the equations, and we find the threshold position at $\mathcal{M}_1 = 0$:

$$\alpha_{m,\text{th}} = \frac{1}{p_M}\left[\alpha_a - \alpha_s p_S + \delta_{am}\left(2 - \frac{\delta_{sm}}{k_{sm}}\right)\right] \approx \frac{1}{p_M}\left[\alpha_a - \alpha_s p_S\right] \tag{1.90}$$
$$\alpha_{a,\text{th}} = \alpha_m p_M + \alpha_s p_S - \delta_{am}\left(2 - \frac{\delta_{sm}}{k_{sm}}\right) \approx \alpha_m p_M + \alpha_s p_S \tag{1.91}$$

From this expression we can deduce that we can shift the threshold to larger α_m if

p_M, p_S↘: $(1 - p_M)$ and $(1 - p_S)$ are the probabilities that a CsrA dimer survives the degradation of an mRNA-CsrA complex and a CsrA-sRNA complex, respectively. If $p_M = 1$ ($p_S = 1$) the regulation is called non-catalytic. If $p_M = 0$ ($p_S = 0$) the regulation is called catalytic, for in this case CsrA acts as a catalyst for the degradation of its binding partners. Then, the threshold value $\alpha_{m,\text{th}}$ is proportional to $1/p_M$, since $1/p_M$ is the number of long mRNA molecules that are degraded along with 1 CsrA dimer.

α_a↗: Increasing α_A leads to a build-up of a larger CsrA buffer that has a greater capability to down-regulate long mRNA expression.

α_s↘: For smaller α_S less sRNA molecules are produced. It follows that less sRNA molecules may interfere with CsrA dimers.

Since equation (1.90) is linear in all production rates, the statement above holds true not only for dimensionless rates α_s, α_a but as well for dimensionful rates α_M, α_A.

1.7 Comparison with Gillespie simulations

We compared the stationary solution of the rate equations with Gillespie simulations. In all Gillespie simulations the starting molecule numbers where set to $M = A = S = 0$.

We observed that the system needs less than $N_{\text{offset}} = 3000$ reactions to relax to equilibrium. We obtained the mean molecule number $\langle M \rangle$ by time averaging the molecule number M over one run between reaction number N_{offset} and $N_{\max} = 1000000$. Each molecule number M was weighted by the waiting time to the next reaction and summed up. At the end, the result was divided by the total time it took between reaction N_{offset} and $N_{\max}$. This can be expressed as:

$$\langle M \rangle = \frac{1}{T} \sum_{i=N_{\text{offset}}}^{N_{\max}-1} \Delta t_i M_i \tag{1.92}$$

$$\text{Var}[M] = \frac{1}{T} \sum_{i=N_{\text{offset}}}^{N_{\max}-1} \Delta t_i M_i^2 - \langle M \rangle^2 \tag{1.93}$$

where $\Delta t_i = t_{i+1} - t_i$ and $T = t_{N_{\max}} - t_{N_{\text{offset}}}$.

The stationary solution M^* might be different from $\langle M \rangle$:

$$\begin{aligned} \dot{\langle M \rangle} &= 0 = \langle \alpha_M \rangle - \langle \delta_M M \rangle - \langle k_M M A \rangle \\ &= \alpha_M - \delta_M \langle M \rangle - k_M \langle [\langle M \rangle + \delta_M][\langle A \rangle + \delta_A] \rangle \\ &= \underbrace{\alpha_M - \delta_M \langle M \rangle - k_M \langle M \rangle \langle A \rangle}_{\text{stationary solution of rate equation}} - k_M [\langle M \rangle \langle \delta_A \rangle + \langle A \rangle \langle \delta_M \rangle + \langle \delta_A \delta_M \rangle] \end{aligned} \tag{1.94}$$

S2 Figure shows very good agreement between the approximative analytical stationary solution of long mRNA abundance and the mean molecule number of mRNA obtained by Gillespie simulations.

1.8 Accounting for additional targets of CsrA

Our study focuses on gene regulation of Colicin E2 release. Therefore, we did not explicitly consider other targets of CsrA (or any component in the *E. coli* cell), although we are aware that CsrA alone can bind to at least over 700 different mRNA targets. The question of how to obtain a simplified biochemical network despite the thousands of different proteins in a living cell, is of very fundamental nature, and remains unsolved. This is particularly critical in our case, since CsrA is a master regulator protein in *E. coli.* However, we still think that it is possible to reduce these system, and want to illustrate, how such a reduction can be done.

In section 1.3 of this Supporting Information, we derived the reduced model from a simplified biochemical network (see S1 Fig). This network comprises five components: The regulator CsrA (A), its target long mRNA (M), the "regulator's regulator" sRNA (S), and the complexes of CsrA with both the long mRNA (C_{MA}) and the sRNA (C_{SA}). For the two types of sRNA (CsrB and CsrC), we derived an effective sRNA, which contains only a single binding site (instead of N binding sites) and thus considerably simplifies the equations (see section 1.3 of the Supporting Information). Employing the effective sRNAs, the biochemical network can be written as the following set of ordinary

differential equations:

$$
\begin{aligned}
\text{CsrA}: \quad & \dot{A} = \alpha_A + k_S^- C_{SA} + k_M^- C_{MA} + \\
& \quad + \delta_{C_{SA}} C_{SA}(1 - p_S) + \delta_{C_{MA}} C_{MA}(1 - p_M) \\
& \quad - \delta_A A - k_M^+ MA - k_S^+ SA, && (1.95) \\
\text{long mRNA}: \quad & \dot{M} = \alpha_M + k_M^- C_{MA} - \delta_M M - k_M^+ MA, && (1.96) \\
\text{long mRNA-CsrA complex}: \quad & \dot{C_{MA}} = k_M^+ MA - k_M^- C_{MA} - \delta_{C_{ma}} C_{MA}, && (1.97) \\
\text{sRNA}: \quad & \dot{S} = N\alpha_S + k_S^- C_{SA} - \delta_S S - k_S^+ SA, && (1.98) \\
\text{sRNA-CsrA complex}: \quad & \dot{C}_{SA} = k_S^+ SA - k_S^- C_{SA} - \delta_{C_{SA}} C_{SA}. && (1.99)
\end{aligned}
$$

These five differential equations describe the temporal change in the abundance of the corresponding quantity. They all contain terms that describe production (α) or degradation (δ) of components, or the formation (k^+) and breaking (k^+) of complexes. Let us shortly recapitulate the biochemical significance of these terms. The first line in the dynamical equation for CsrA (eq. (1.95)) comprises the rate of CsrA production (α_A), and two terms accounting for the increase in CsrA due to CsrA-sRNA- and CsrA-mRNA-complexes breaking up, respectively. The next line contains two terms describing the CsrA increase by the degradation of these two complexes, and include the parameters p_M and p_S, which describe the probability for CsrA to be co-degraded with the complex. Finally, the last line describes terms which reduce CsrA abundance: CsrA decreases either by degradation of CsrA (δ_A), or by forming complexes with long mRNA or sRNA, respectively. The equations for long mRNA and sRNA, eq. (1.96) and (1.98), describe analogous biochemical processes. As the formation of a complex means a decrease in the abundance of the respective complex partners, we find in the equations of the complexes, eq. (1.97) and (1.99), that terms with positive sign in the dynamical equations of A, M or S appear with negative sign the equations for complexes, and vice versa.

In order to account for a new target, we assume that its qualitative behavior is that of long mRNA. This means that in the model its differential equation has the very same structure as that for the long mRNA, but of course with rate parameters specific to the corresponding target. As it would be very unhandy to add over 700 targets to the model, we introduce a single, effective target, T. This additional effective target is an "average" mRNA target, which forms complexes with CsrA. Therefore, accounting for such an effective target adds dynamic equations for the abundance of the target as well as for its complexes,

$$
\begin{aligned}
\text{eff. target}: \quad & \dot{T} = \alpha_T + k_T^- C_{TA} - k_T^+ AT - \delta_T T && (1.100) \\
\text{target-CsrA complex}: \quad & \dot{C}_{TA} = k_T^+ TA - k_T^- C_{TA} - \delta_{C_{TA}} C_{TA}, && (1.101)
\end{aligned}
$$

and also adds new terms to the dynamic equation for CsrA, eq. (1.95):

$$
\text{CsrA}: \quad \dot{A} = [\text{r.h.s. of (1.95)}] - k_T^+ TA + k_T^- C_{TA} + \delta_{C_{TA}} C_{TA}(1 - p_T). \qquad (1.102)
$$

As stated above, the structure of its terms is analogous to those found in the dynamics of the long mRNA: The effective target is produced at rate α_T and degraded at rate $\delta_T T$. Note that α_T is chosen such that the target abundance in the cell matches the combined abundance of the 700 different targets. As with long mRNA, the abundances of A and T get reduced by the formation of CsrA-target-complex ($-k_T^+ TA$), and increased once these complexes either break apart ($+k_T^- C_{TA}$) or get degraded ($+\delta_{C_{TA}} C_{TA}(1 - p_T)$). In the equation for the CsrA-target-complexes, the last three rates appear again with opposite signs.

Eqs.(1.96)-(1.102) now define our initial biochemical network, extended with an effective additional target and its complex with CsrA. We proceed by first considering this system in the steady state. From this state, we can calculate the component abundances. In a second step, we make the simplifying assumptions that all complexes and, in a third step, also the target abundance equilibrate fast compared to other components of the reduced model. This fast-equilibrium-assumption eliminates the affected biochemical processes, and allows us to finally reduce the model to three components.

Steady State. In order to compare the abundances predicted by the extended biochemical network with experimental data, we start by considering the steady state of the system. The steady state is defined as the state, in which no abundance is subject to changes with time. It is obtained by setting the left hand sides of eqs.(1.96)-(1.102) to zero (i.e. $\dot{A} = 0, \dot{M} = 0, ...$). We begin with the equations for the complexes, eqs. (1.97), (1.99) and (1.101). For C_{MA} (eq. (1.97)), we get from $\dot{C} = 0$,

$$C_{MA} = \frac{k_M^+ \cdot M \cdot A}{k_M^- + \delta_{C_{MA}}} = \frac{k_M \cdot M \cdot A}{\delta_{C_{MA}}}, \tag{1.103}$$

where we introduced the effective binding parameters

$$k_M := \frac{k_M^+ \delta_{C_{MA}}}{k_M^- + \delta_{C_{MA}}}. \tag{1.104}$$

As the equations of the complexes, eqs. (1.97), (1.99) and (1.101), have all the very same structure, we can find equations and effective parameters for the sRNA/CsrA- and target/CsrA-complexes analogously. Taken together, these equations read

$$C_{MA} = \frac{k_M \cdot M \cdot A}{\delta_{C_{MA}}}, \qquad k_M := \frac{k_M^+ \delta_{C_{MA}}}{k_M^- + \delta_{C_{MA}}}, \tag{1.105}$$

$$C_{SA} = \frac{k_S \cdot S \cdot A}{\delta_{C_{SA}}}, \qquad k_S := \frac{k_S^+ \delta_{C_{SA}}}{k_S^- + \delta_{C_{SA}}}, \tag{1.106}$$

$$C_{TA} = \frac{k_T \cdot T \cdot A}{\delta_{C_{TA}}}, \qquad k_T := \frac{k_T^+ \delta_{C_{TA}}}{k_T^- + \delta_{C_{TA}}}. \tag{1.107}$$

Inserting these equations to the (steady state) differential equations for A, S, M and T, we obtain an set of coupled equations that is independent of the complex abundances:

$$0 = \alpha_A - \delta_A A - k_M p_M M \cdot A - k_S p_S S \cdot A - k_T p_T A \cdot T, \tag{1.108}$$

$$0 = \alpha_M - \delta_M M - k_M M \cdot A, \tag{1.109}$$

$$0 = N\alpha_S - \delta_S S - k_S S \cdot A, \tag{1.110}$$

$$0 = \alpha_T - \delta_T T - k_T T \cdot A. \tag{1.111}$$

These four equations describe the steady state of the free components A, S, M and T. Note that by employing eqs. (1.105)-(1.107), we were able to combine for each component complex degradation and (un)binding of the complex partners to an effective "coupled degradation" term, e.g. $-k_M p_M M A$ for long mRNA. This step reduces the complexity: The equations for M, S and T now contain only three terms, one each for production (α), degradation (δ) and complex formation with CsrA (k). The dynamic equation for CsrA has the same structure, but a special coupled degradation term: As CsrA forms complexes with each of the three other components (M, S and T), it also has three coupled degradation terms.

Solving our system of equations, eqs. (1.108)-(1.111) numerically allows us to calculate the steady state abundances of the system. To this end, we need to estimate the production, degradation, and binding rates of all the components. We motivate our estimations in chapter 2. Moreover, we assumed $p_M = p_S = p_T = 1$, as CsrA dimers, even if they survive complex degradation, are unlikely to form a complex again after prolonged binding. More specifically, we used the following parameters (in the units molecules/min, 1/min, 1/(molecules · min) for α, γ, k, respectively):

$$\text{M:} \quad \alpha_M = 1 \quad \delta_M = 0.04 \quad k_M = 0.5 \quad p_M = 1 \quad \delta_{C_M} = \delta_M \tag{1.112}$$
$$\text{S:} \quad N\alpha_S = 57.5 \quad \delta_S = 0.023 \quad k_S = 0.5 \quad p_M = 1 \quad \delta_{C_S} = \delta_S \tag{1.113}$$
$$\text{T:} \quad \alpha_T = 350 \quad \delta_T = 0.04 \quad k_T = 0.5 \quad p_T = 1 \quad \delta_{C_T} = \delta_T \tag{1.114}$$
$$\text{A:} \quad \alpha_A = 408.45 \quad \delta_A = 0.00007 \tag{1.115}$$

Note that the production rates α of the components are unknown (see also chapter 2), and are thus treated as free parameters. We chose them such that the numerical solution of eqs. (1.108)-(1.111) with the other parameters in eqs. (1.112)-(1.115) results in

$$M = 0.01, \quad S = 0.29, \quad T = 1.77, \quad A = 395.45,$$
$$C_{MA} = 24.99, \quad C_{TA} = 8748.23, \quad C_{SA} = 2499.71.$$

We find that our model consistently predicts not only the abundance of free CsrA (A) as found by Taniguchi et al. [7](474 ± 191 free CsrA molecules), but also its total abundance $A + C_{MA} + C_{TA} + C_{SA}$ and the sRNA ratio, which are given by Gudapathy et al. (11.000-33.000 CsrA molecules in total, 16-32% bound to sRNA [8]). This shows that the abundances in our model reconcile with abundances found in experiments. Moreover, the model extended with the effective targets produced the same abundance of free CsrA as our reduced model. From this we learn that it is indeed justifiable to use a reduced model, which does not account for all possible targets, as the abundance of free CsrA is sufficient to describe our specific regulatory system.

Dynamics. The next steps, which reduce the number of components in the model, are more difficult, and require us to make assumptions on the speed of equilibration of a subset of biochemical processes. Specifically, we assume fast complex equilibration (i.e. $\dot{C}_{SA} = 0, ...$). This assumption is well established in the literature (see, for instance, [9]), and has already been employed in our derivation of the reduced model. It allows us to use eqs. (1.105)-(1.107):

$$C_{MA} = \frac{k_M \cdot M \cdot A}{\delta_{C_{MA}}},$$
$$C_{SA} = \frac{k_S \cdot S \cdot A}{\delta_{C_{SA}}},$$
$$C_{TA} = \frac{k_T \cdot T \cdot A}{\delta_{C_{TA}}},$$

This yields the following set of equations

$$\dot{A} = \alpha_A - \delta_A A - k_M MA - k_S SA - k_T AT, \tag{1.116}$$
$$\dot{M} = \alpha_M - \delta_M M - k_M MA, \tag{1.117}$$
$$\dot{S} = N\alpha_S - \delta_S S - k_S SA, \tag{1.118}$$
$$\dot{T} = \alpha_T - \delta_T T - k_T TA. \tag{1.119}$$

Note that the right hand side of these equations is identical to the right hand side of the steady state equations, eqs.(1.96)-(1.102). These four equations describe the dynamics of our reduced model (as presented in our paper), interacting with a second, effective target for CsrA.

So far, we have showed how it is possible to account for additional targets in a cell in the form of a single, effective target, and derived a reduced four-component model, eqs. (1.116)-(1.119). In order to work with these equations, we must specify the dynamics of the effective target. However, the corresponding rates for most targets are not known, and can only be estimated roughly. It is therefore not useful to explicitly account for these targets in the model. In the following paragraph, we will use the aforementioned rough estimates to reduce eqs. (1.116)-(1.119) back to our three-component model, as the additional terms for the target turn out to be constant above a threshold value of A.

Elimination of the Target Dynamics. In order to eliminate T from eqs. (1.116)-(1.119), we proceed analogous to the elimination of the complexes and make the additional assumption that also the target abundance equilibrates fast. This means that we assume $\dot{T} = 0$ in eq. (1.119), and, just as with the complexes (see, e.g., eq. (1.103)), solve for the target abundance:

$$T = \frac{\alpha_T}{\delta_T + k_T A}. \tag{1.120}$$

We then insert this solution in the differential equation for A, eq. (1.116):

$$\dot{A} = \alpha_A - \delta_A A - k_M M A - k_S S A - k_T \cdot A \cdot \frac{\alpha_T}{\delta_{CT} + k_T A}. \tag{1.121}$$

The equation for A is now independent of T, and eqs. (1.121),(1.117) and (1.118) comprise a closed system of differential equations for three components (just as in our reduced model presented in our main text). If we compare it to our reduced model, we find that the models differ only by a single degradation term in the equation for CsrA. The term reads

$$-k_T \cdot A \cdot \frac{\alpha_T}{\delta_{CT} + k_T A} = -A \cdot \frac{\alpha_T}{\frac{\delta_{CT}}{k_T} + A}, \tag{1.122}$$

and is special for two reasons: First, it is the only term that contains the parameters for the effective targets, and thus describes their influence on the dynamics. Second, its dependence on the parameter A is more complex than for the other terms in eq. (1.121), as it has it has a Langmuir-like dependence on A. Because of the Langmuir functional form, the ratio of δ_{CT} and k_T determines whether the term depends on A or not: If δ_{CT}/k_T is significantly larger than A, it dominates the denominator in eq. (1.122), and the term becomes linearly dependent on A. In the opposite case, if A dominates the denominator, it cancels with the linear A-dependence, rendering the term constant. These two limiting scenarios can be summarized as follows:

$$\frac{\delta_{CT}}{k_T} \ll A: \qquad A \cdot \frac{\alpha_T}{\frac{\delta_{CT}}{k_T} + A} \approx A \cdot \frac{\alpha_T}{A} = \alpha_T, \tag{1.123}$$

$$\frac{\delta_{CT}}{k_T} \gg A: \qquad A \cdot \frac{\alpha_T}{\frac{\delta_{CT}}{k_T} + A} \approx A \cdot \frac{\alpha_T}{\frac{\delta_{CT}}{k_T}} = A \cdot \frac{\alpha_T k_T}{\delta_{CT}}. \tag{1.124}$$

If the parameter sets used in our simulations fall into one of the two limiting scenarios, we could approximate the Langmuir-like term by either eq.(1.123) or (1.124). This

would then simplify the analysis of the equations, and allows us to absorb the term eq. (1.122) into an effective rate of production or degradation, respectively.

Using the parameters defined in eq. (1.114), we find $\frac{\delta_{CT}}{k_T} = 0.08$. For steady state calculations before an SOS signal, that is, when $A \gg 1$, we can thus assume $\frac{\delta_{CT}}{k_T} \ll A$. This means that eq.(1.123) can be applied, and eq. (1.122), becomes the constant α_T. We insert it to eq. (1.121) to get:

$$\begin{aligned}
\dot{A} &= \alpha_A - \delta_A A - k_M M A - k_S A S - k_T \cdot A \cdot \frac{\alpha_T}{\delta_{CT} + k_T A} && (1.125)\\
&\approx \alpha_A - \delta_A A - k_M M A - k_S A S - \alpha_T && (1.126)\\
&= (\alpha_A - \alpha_T) - \delta_A A - k_M M A - k_S A S && (1.127)\\
&= \alpha_{A,\text{eff}} - \delta_A A - k_M M A - k_S A S. && (1.128)
\end{aligned}$$

In these steps, we approximated the Langmuir-like term by the constant limiting case, as described above. We then eliminated this now constant term by adding it to the (also constant) production rate α_A, thus defining a new, effective production rate

$$\alpha_{A,\text{eff}} = \alpha_A - \alpha_T.$$

Calculating this effective production rate from the parameters defined in eqs. (1.115) and (1.114), we get $\alpha_{A,\text{eff}} = 58.45$. If we use this value to numerically solve the steady state eqs. (1.128),(1.117) and (1.118), we find that $\alpha_{A,\text{eff}}$ does not reproduce the correct steady state abundances. However, to get the correct values, we have to slightly increase this value to 58.52. This slight difference stems from the approximation of the Langmuir-like term.

With the correction, we get for our three-component system

$$\begin{aligned}
M &= 0.01, & S &= 0.30, & A &= 386.44,\\
C_{MA} &= 24.99, & C_{SA} &= 2499.7,
\end{aligned}$$

which matches the abundances found in the steady state solution with the targets again very well.

In summary, we derived the three-component system from our main text,

$$\begin{aligned}
\dot{M} &= \alpha_M - \delta_M M - k_M M A,\\
\dot{A} &= \alpha_A - \delta_A A - k_M p_M M A - k_S p_S A S,\\
\dot{S} &= N\alpha_S - \delta_S S - A k_S S,
\end{aligned}$$

from our initial biochemical network, eqs. (1.95)-(1.99), which now also accounted for additional targets, eqs.(1.100) and (1.101). We found, that the following set of rate parameters (in the units molecules/min, 1/min, 1/(molecules · min) for α, γ, k, respectively):

M:	$\alpha_M = 1$	$\delta_M = 0.04$	$k_M = 0.5$	$p_M = 1$	$\delta_{C_M} = \delta_M$
S:	$N\alpha_S = 57.5$	$\delta_S = 0.023$	$k_S = 0.5$	$p_M = 1$	$\delta_{C_S} = \delta_S$
A:	$\alpha_A = 58.52$	$\delta_A = 0.00007$			

the model is able to reproduce experimentally observed abundances.

Chapter 2

Biological parameter values in post-transcriptional regulation

The goal of this chapter is to find meaningful parameter values. We follow a three step procedure. First, literature is searched for experimental measurements. Second, if there are no experimental measurements, we will estimate the range of parameter values by looking at similar regulation systems in bacteria. Third, considering the explicit biological processes involved in expression, we will find rough estimates for parameter values and we will check if these values agree with the biological range found in step two.

2.1 Experimental values

At 37°C the half-life of small RNAs CsrB and CsrC are 1.6 min and 4.1 min, respectively [2]. Since the relation between degradation rates and half-life is $\delta = {}^{1}/_{\tau} = {}^{\ln 2}/_{t_{1/2}}$, we find $\delta_{\mathrm{CsrB}} \approx 0.43\ \mathrm{min}^{-1}$ and $\delta_{\mathrm{CsrC}} \approx 0.17\ \mathrm{min}^{-1}$. However, these values have been measured in experimental conditions that suggest the presence of CsrD, which was shown to be responsible for the degradation of the sRNAs CsrB and CsrC. Recent studies show that CsrB/C decay is activated by the presence of glucose, because glucose leads finally to activation of CsrD [10]. Another recent study demonstrated, that only the unphosphorylated form of EIIAglc (the glucose specific permease of the PTS system) is able to bind to CsrD and activate CsrB/C degradation [11]. In this regard, [12] found that in glucose media, EIIA is unphosphorylated, but phosphorylated in glycerol media. As we want to compare the results of our model to experiments which employ glycerol as the only carbon source, CsrD will not be activated. This implies that we can consider the half-life of the sRNA to be about 30 min [13], which corresponds to $\delta_S = 0.023\ \mathrm{min}^{-1}$.

Long mRNA decays with a half-life of $18 \pm 1.5\,\mathrm{min}$ [14], which leads to $\delta_M \approx 0.04\ \mathrm{min}^{-1}$.

For proteins in *E. coli* we know that "[o]nly a limited portion of the cellular protein is subject to rapid degradation. It decays with a half-life of approximately 1 hour and constitutes 2 to 7% of the total cellular protein" [15]. This classification was refined one year later [16]:

1. $2\% - 7\%$ of all proteins in *E. coli* degrade quickly with a half-life of approximately one hour, meaning $\delta_{\text{fast}} \approx 0.012\ \text{min}^{-1}$.
2. The remainder, i.e. $93\% - 98\%$ of all proteins in *E . coli*, degrades
   (a) under starvation at a rate of $2.5\% - 6\%$ of proteins per hour. Since $N(t) = N_0 e^{-\delta t}$, it follows that $\delta_{\text{starve}} = -\ln(0.98 - 0.935)\ \text{hr}^{-1} = (0.0034 - 0.001)\ \text{min}^{-1}$.
   (b) without starvation at a rate of $0.2\% - 0.6\%$ of proteins per hour. It follows that $\delta_{\text{slow}} = (0.000034 - 0.0001)\ \text{min}^{-1}$.

Since we are not interested in conditions under starvation, we have to choose either the degradation rate of fast degrading proteins with $\delta_{\text{fast}} \approx 0.01\ \text{min}^{-1}$, or that of slowly degrading proteins with $\delta_{\text{slow}} \approx 0.00007\ \text{min}^{-1}$. Since CsrA is generally described as very stable [13], we assume $\delta_A = 0.00007\ \text{min}^{-1}$.

In summary, we set the degradation rates as

$\delta_M = 0.04\ \text{min}^{-1}$	$\delta_A = 0.00007\ \text{min}^{-1}$	$\delta_S = 0.023\ \text{min}^{-1}$

2.2 Estimations from similar system

In [6] a cyanobacterial iron stress response was analyzed and analytic calculations were fitted to experimental data. This lead to an estimate of complex binding parameters. The best-fit parameters for an up-regulated system are as follows:

$$k = \frac{k_{\text{on}}\delta_C}{k_{\text{off}} + \delta_C} \approx \frac{k_{\text{on}}\delta_C}{k_{\text{off}}} \approx 4.4\ \text{nM}^{-1}\text{min}^{-1}.$$

In [5] the target gene *sodB* was regulated by an sRNA, RyhB, that is involved in iron homeostasis of *E. coli*. The complex binding parameters were estimated to

$$k = 0.02\ \text{nM}^{-1}\text{min}^{-1}.$$

The estimates for complex binding parameters deviate in the two different systems by two orders of magnitudes. We will use these results as the biological range for these parameters in our model of post-transcriptional regulation. We choose the mean order of magnitude and take:

$$\boxed{k_M = k_S = 0.5\ \text{min}^{-1}\text{molecule}^{-1}.}$$

Cooperative Binding. We are aware of the fact that some studies (like dscussed in [17, 18]) suggest that CsrB and CsrC are subject to positive cooperative binding, that is, an increase in binding affinity of a sRNA the more CsrA molecules bind to it. However, we were not able to find reliable quantitative data, which would clearly show that our assumption of fast complex equilibration is void. Indirect ways of analyzing the binding rates, particularly the measurement of K_D values, produce highly varying results, depending on the particular experimental condition used [19, 20]. Since clear evidence for highly cooperative binding interfering with our assumptions is missing, we did not include this phenomenon in our model. In the case that future studies would show that cooperative binding effects of CsrA to sRNA are indeed crucial, our model is still valid, but has to be slightly extended: As the CsrA-sRNA-complexes cannot be considered to equilibrate fast anymore, their abundance C_{SA} must be included explicitly in the model. This would turn the three-component-model to a four-component one.

2.3 Biological estimations

Conversion of units

First, we have to find a conversion between the unit nanomolar (nM), as found in experimental papers, and molecules per cell, as we have used for our estimations. The cell volume of *E. coli* is $V_{EC} = (0.6 - 0.7)\mu m^3$ [21]. Therefore,
$1\text{nM} = 10^{-9}\frac{\text{mol}}{\text{dm}^3} = 10^{-9}\frac{0.65}{1}\frac{6.02\cdot 10^{23}\text{ molecules}}{0.65\cdot 10^{15}\mu m^3} \approx \frac{0.4\text{ molecules}}{0.65\mu m^3} = \frac{0.4\text{ molecules}}{V_{EC}}$.

Production rates

The production rates of CsrA, the sRNAs and the long mRNA, α_A, α_S and α_M, have not been measured, and are thus unknown. In order to obtain plausible values, we fit them such that our model produces component abundances that are found in the literature.

For CsrA, Taniguchi [7] finds an abundance of free molecules of 474 ± 191 per cell. In the reduced model, this value is reached in the steady state if we set $\alpha_A = 58.52\ ^{\text{molecules}}/_{\text{min}}$. We show in section 1.8 of this Supporting Information, that this number is in good agreement with an extended model, which also accounts for additional targets of CsrA and correctly reproduces the total CsrA abundance in the cell.

For sRNA, Gudapathy et al. [8] find an abundance of about $S_{\text{exp}} = 250$ CsrB molecules per cell. Moreover, they assume that all of these molecules have formed a complex with CsrA molecules (that is, there is no free sRNA in the system), and that for each sRNA all binding sites are occupied with CsrA. The CsrB molecule is known to have approximately 22 binding sites for CsrA, with $N \approx 10$ CsrA dimers being attached on average [1, 22]. Since our model uses an effective sRNA with only a single binding site, we have to fit the sRNA production rate, $N\alpha_S$, such that our model produces the N-fold amount of sRNAs compared to the abundance found in experiments. In order to get $N \cdot S_{\text{exp}} = 10 \cdot 250$ sRNA complexes, we need to set $N\alpha_S = 57.5\ ^{\text{molecules}}/_{\text{min}}$.

For the long mRNA, the abundance in the cell has not been measured yet. Since the gene of long mRNA (2335 nucleotides [23]) is about one order of magnitude larger than the ones of CsrA (183 nucleotides [23]) and the sRNAs (369 nucleotides and 245 *nt* [23]), the transcription of long mRNA will take longer. Consequently, we assumed α_M to be significantly smaller than the production rates of CsrA and sRNA. In our model, we assumed it to be $\alpha_M = 1$.

In summary, we defined the following production rates for long mRNA, CsrA and sRNA:

$\alpha_M = 1\ ^{\text{molecules}}/_{\text{min}}$	$\alpha_A = 58.52\ ^{\text{molecules}}/_{\text{min}}$	$\alpha_S = 57.5\ ^{\text{molecules}}/_{\text{min}}$

Plasmid numbers

Previous studies showed that in a single *E. coli* cell there are approximately $n_{\text{sos}} = 20$ copies of the plasmid containing the colicin operon. The variation in the colcin plasmid number from cell to cell is caused by colicin E2 plasmids being steadily replicated in the cell by a rolling circle replication mechanism. The plasmid copy number enters our

model via the total (per cell) production rate of long mRNA (α_M) as a multiplicative factor: $\alpha_M = \alpha_{M_l} \cdot (n_{\text{sos}} - B_{\text{SOS}})$ (where B_{SOS} denotes the number of plasmids with respressed colicin promoter). Therefore, changes in the plasmid copy number affect our model by either increasing (fewer plasmids) or decreasing (more plasmids) the delay between SOS signal and lysis. However, since most colicin promoters are repressed, even during an SOS signal, the consequences of this effect are limited, compared to changes in the rate parameters. To show the effects of varying plasmid copy numbers, we briefly discuss the lysis time distribution of a population, in which the plasmid copy number is Poisson-distributed with mean $n_{\text{sos}} = 20$ (see S4 Fig B). Compared to its counterpart with fixed n_{sos}, S4 Fig A, we find that the distribution in S4 Fig B is wider. This is due to the effects of variation in n_{sos} we described above: As the population contains cells with plasmid levels both above and below the average, the distribution gets shifted in both directions. However, the comparison with S4 Fig A also shows that the widening of the distribution is rather weak, and that the overall shape of the distribution is largely conserved. This illustrates that variations in plasmid copy number affect the lysis time distribution only weakly. Moreover, the replication of plasmids is the only mechanism that affects their copy number, and happens much slower than any other process considered by our model. Hence, the effect of variation in plasmid copy number on lysis time distributions is expected to be only minor. In order to keep the focus on effects happening on the timescale of SOS responses, we kept the number of colicin plasmids constant, and chose their abundance to be the average value.

Chapter 3

Gillespie simulations

We define the following notation:

- R, Le, Col, L: number of RecA proteins, LexA dimers, Colicin proteins and lysis proteins.
- $M_r M_l, M_s, M$: number of *lexA*, *recA*, short mRNAs and long mRNAs.
- S: number of effective sRNAs with one CsrA binding site.
- $B_r B_l, B_{\text{sos}}$: number of LexA dimers bound to the *lexA*, *recA* and SOS promoter.
- $\alpha_{M_r}, \alpha_{M_l}, \alpha_R, \alpha_{Le}, B_{\text{sos}}, \alpha_{M_s}, \alpha_M, \alpha_A, \alpha_S, \alpha_L$: Production rates ($\alpha$) of the component denoted by the subscript.
- $\delta_{M_r}, \delta_{M_l}, \delta_{Le}, \delta_R, \delta_{M_s}, \delta_M, \delta_A, \delta_S, \delta_L$: Degradation rate of the component denoted by the subscript.
- $k_r^+, k_l^+, k_{sos}^+, k_r^-, k_l^-, k_{sos}^-$: Binding rates (+) and unbinding rates (−) of LexA dimers to *recA*, *lexA* and SOS promoter sites. The subscript denotes the component.
- k_M, k_S: coupled degradation parameters for the complexes of mRNA and sRNA, respectively
- c_p: Rate of LexA auto-cleavage due to RecA protein.
- n_{sos}: number of ColE2 plasmids.
- $1 - p_M$: Probability of CsrA dimers surviving degradation of sRNA-CsrA complexes.
- $1 - p_S$: Probability of CsrA dimers surviving degradation of mRNA-CsrA complexes.

3.1 Rate equations

With the defined interaction scheme and notation we are now able to introduce the rate equations used by Shimoni [24]:

$$\dot{M_r} = \alpha_{M_r}(1 - B_r) - \delta_{M_r} M_r \tag{3.1}$$

$$\dot{M_l} = \alpha_{M_l}(1 - B_l) - \delta_{M_l} M_l \tag{3.2}$$

$$\dot{R} = \alpha_R M_r - \delta_R R \tag{3.3}$$

$$\begin{aligned}\dot{Le} = {} & \alpha_{Le} M_l - \delta_{Le} Le - k_l^+ (1 - B_l) Le + k_l^- B_l - k_r^+ (1 - B_r) Le + k_r^- B_r \\ & - k_{sos}^+ (1 - B_{\text{sos}}) Le + k_{sos}^- B_{\text{sos}} - c_p R Le\end{aligned} \tag{3.4}$$

$$\dot{B_r} = k_r^+ (1 - B_r) Le - k_r^- B_r \tag{3.5}$$

$$\dot{B_l} = k_l^+ (1 - B_l) Le - k_l^- B_l \tag{3.6}$$

$$\dot{B_{\text{sos}}} = k_{sos}^+ (n_{\text{sos}} - B_{\text{sos}}) Le - k_{sos}^- B_{\text{sos}} \tag{3.7}$$

$$\dot{M_s} = \alpha_{M_s}(n_{\text{sos}} - B_{\text{sos}}) - \delta_{M_s} M_s \tag{3.8}$$

$$\dot{M} = \alpha_{M_l}(n_{\text{sos}} - B_{\text{sos}}) - \delta_M M - k_M M A \tag{3.9}$$

$$\dot{A} = \alpha_A - \delta_A A - k_M p_M M A - k_S p_S A S \tag{3.10}$$

$$\dot{S} = \alpha_S - \delta_S S - A k_S S \tag{3.11}$$

3.2 Gillespie simulations

From the rate equations (3.1)-(3.11) we set up a Gillespie simulation [25] with the following reactions:

1. $M_r \xrightarrow{\alpha_{M_r}(1-B_r)} M_r + 1$
2. $M_l \xrightarrow{\alpha_{M_l}(1-B_l)} M_l + 1$
3. $R \xrightarrow{\alpha_R M_r} R + 1$
4. $Le \xrightarrow{\alpha_{Le} M_l} Le + 1$
5. $M_r \xrightarrow{\delta_{M_r} M_r} M_r - 1$
6. $M_l \xrightarrow{\delta_{M_l} M_l} M_l - 1$
7. $R \xrightarrow{\delta_R R} R - 1$
8. $Le \xrightarrow{\delta_{Le} Le} Le - 1$

9. $Le, B_r \xrightarrow{k_r^+(1-B_r)Le} Le - 1, B_r + 1$
10. $Le, B_l \xrightarrow{k_l^+(1-B_l)Le} Le - 1, B_l + 1$
11. $Le, B_r \xrightarrow{k_r^- B_r} Le + 1, B_r - 1$
12. $Le, B_l \xrightarrow{k_l^- B_l} Le + 1, B_l - 1$
13. $Le \xrightarrow{c_p R Le} Le - 1$
14. $Le, B_{\text{sos}} \xrightarrow{k_{sos}^+(n_{\text{sos}}-B_{\text{sos}})Le}$ $Le - 1, B_{\text{sos}} + 1$
15. $Le, B_{\text{sos}} \xrightarrow{k_{sos}^- B_{\text{sos}}} Le + 1, B_{\text{sos}} - 1$

16. $M_s \xrightarrow{\alpha_{M_s}(n_{\text{sos}}-B_{\text{sos}})} M_s + 1$
17. $M_s \xrightarrow{\delta_{M_s} M_s} M_s - 1$
18. $M \xrightarrow{\alpha_{M_l}(n_{\text{sos}}-B_{\text{sos}})} M + 1$
19. $M \xrightarrow{\delta_M M} M - 1$

20. $A \xrightarrow{\alpha_A} A+1$

21. $A \xrightarrow{\delta_A A} A-1$

22. $S \xrightarrow{\alpha_S} S+1$

23. $S \xrightarrow{\delta_S S} S-1$

24. $(M,A) \xrightarrow{k_M p_M MA} (M-1, A-1)$

25. $(M,A) \xrightarrow{k_M(1-p_M)MA} (M-1, A)$

26. $(A,S) \xrightarrow{k_S p_S AS} (A-1, S-1)$

27. $(A,S) \xrightarrow{k_S(1-p_S)AS} (A, S-1)$

The parameter values are shown in S1 Table. The values from literature in this table were taken from [2, 14–16, 24, 26]. Estimated parameter values were chosen according to [5, 6, 24]. For the transcription rates of long mRNA, CsrA and sRNA we calculated a rough estimate using the transcription rate of RNA polymerase [27] and the length of the individual genes [23], taking into account the number of Colicin plasmids, CsrA binding sites on CsrB, and the translational burst size for CsrA. All parameters are given in the unit of molecules per cell and minute.

Chapter 4

Linear noise approximation

4.1 Definitions

The state vector $\vec{x} = (X_1, X_2, \ldots, X_N)^T$ gives the copy numbers of the N components involved.

There are M reactions with rates $\vec{W}(\vec{x}) = (W_1(\vec{x}), W_2(\vec{x}), \ldots, W_M(\vec{x}))^T$.

The matrix $\mathcal{A}$ with components a_{ij} gives the change in copy number of component i following reaction j.

4.2 Master equation and rate equation

With the definitions above the master equation is given by:

$$\frac{d}{dt}P(\vec{x}, t) = \sum_{j=1}^{M} [W_j(\vec{x} - \vec{a}_j)P(\vec{x} - \vec{a}_j, t) - W_j(\vec{x})P(\vec{x}, t)] \tag{4.1}$$

The Master equation 4.1 gives rise to the time evolution of the first moment:

$$\langle \dot{\vec{x}} \rangle = \langle \mathcal{A}\vec{W}(\vec{x}) \rangle \approx \mathcal{A}\vec{W}(\langle \vec{x} \rangle) \tag{4.2}$$

In the last step we have neglected correlations. If all reaction rates in vector $\vec{W}$ were linear, an equal sign would hold true. Equation (4.2) with neglected correlations is the deterministic rate equation of the system.

4.3 Kramers-Moyal expansion and van Kampen's expansion

The master equation (4.1) is a set of N_x coupled ordinary differential equations (ODEs), where N_x is the number of states in the system. There is a large number of states, since each set of copy numbers corresponds to one individual state. This makes it very hard

to acquire useful information directly from the master equation. A master equation is often approximated by a Kramers-Moyal expansion, which converts the set of N_x coupled ODEs to one partial differential equation of order $i_{\max}$:

$$\frac{d}{dt}P(\vec{x},t) = \partial_t P(\vec{x},t) \approx \sum_{i=1}^{i_{\max}} \left(\frac{(-1)^i}{i!} \prod_{j=1}^{i} (\partial x_{k_j}) \left[\prod_{j=1}^{i} (a_{k_j l}) W_l(\vec{x}) P(\vec{x},t) \right] \right) \tag{4.3}$$

For the sake of concise notation, we define $\vec{\mathcal{X}} = \langle \vec{x} \rangle$, so $\partial_t \vec{\mathcal{X}} = \mathcal{A}\vec{W}(\vec{\mathcal{X}})$. Next, we introduce a new random variable $\vec{\xi}$, which gives the fluctuations around the deterministic trajectory given by the rate equations:

$$\vec{x} = \vec{\mathcal{X}} + \vec{\xi} \qquad \text{with } \vec{\xi} = \mathcal{O}\left(\sqrt{|\vec{x}|}\right) \tag{4.4}$$

It is important that fluctuations scale with the square root of the mean, because van Kampen's expansion is only valid if fluctuations are in the vicinity of the deterministic rate equation. Contrary to the van Kampen expansion typically found in textbooks [4, 28], we neglect the system size parameter Ω used for scaling arguments at this point. The reasons will become clear in section 4.6.

The probability density in the new random variable $\vec{\xi}$ relates to the probability density in the random variable $\vec{x}$ as

$$\pi(\vec{\xi},t) = P(\vec{x},t) = P(\vec{\mathcal{X}}(t) + \vec{\xi},t). \tag{4.5}$$

Consequently, we find:

$$\begin{aligned} \partial_t \pi(\vec{\xi},t) &= \partial_{x_i} P(\vec{x},t) \frac{dx_i(t)}{dt} + \partial_t P(\vec{x},t) \\ &= \partial_{x_i} P(\vec{x},t) \frac{d\mathcal{X}_i(t)}{dt} + \sum_{i=1}^{i_{\max}} \left(\frac{(-1)^i}{i!} \prod_{j=1}^{i} (\partial x_{k_j}) \left[\prod_{j=1}^{i} (a_{k_j l}) W_l(\vec{x}) P(\vec{x},t) \right] \right) \\ &= \partial_{\xi_i} \pi(\vec{\xi},t) a_{ij} W_j(\vec{\mathcal{X}}) \\ &\quad + \sum_{i=1}^{i_{\max}} \left(\frac{(-1)^i}{i!} \prod_{j=1}^{i} (\partial \xi_{k_j}) \left[\prod_{j=1}^{i} (a_{k_j l}) W_l(\vec{\mathcal{X}} + \vec{\xi}) \pi(\vec{\xi},t) \right] \right) \\ &= -\partial_{\xi_i} \left(\left[a_{il} W_l(\vec{\mathcal{X}} + \vec{\xi}) - a_{il} W_l(\vec{\mathcal{X}}) \right] \pi(\vec{\xi},t) \right) \\ &\quad + \sum_{i=2}^{i_{\max}} \left(\frac{(-1)^i}{i!} \prod_{j=1}^{i} (\partial \xi_{k_j}) \left[\prod_{j=1}^{i} (a_{k_j l}) W_l(\vec{\mathcal{X}} + \vec{\xi}) \pi(\vec{\xi},t) \right] \right). \end{aligned} \tag{4.6}$$

To perform these calculations, we used several times the equality

$$\begin{aligned} \partial_{\xi_i} \left[\pi(\vec{\xi},t) f(\vec{x}(\vec{\xi})) \right] &= \partial_{\xi_i} \left[P(\vec{x}(\vec{\xi}),t) f(\vec{x}(\vec{\xi})) \right] = \partial_{x_j} \left[P(\vec{x},t) f(\vec{x}) \right] \underbrace{\frac{dx_j}{d\xi_i}}_{\delta_{i,j}} \\ &= \partial_{x_i} \left[P(\vec{x},t) f(\vec{x}) \right]. \end{aligned} \tag{4.7}$$

The basic assumption here is that fluctuations $\vec{\xi}$ around the mean $\vec{\mathcal{X}}$ are expected to scale with the square root of the mean as denoted in equation (4.4). It follows that for large $\vec{\xi}$ and a sufficiently smooth reaction rate vector, we may perform a Taylor expansion:

$$W_l(\vec{\mathcal{X}} + \vec{\xi}) = \sum_{u=0}^{\infty} \frac{1}{u!} \prod_{v=1}^{u} (\partial_{\mathcal{X}_{k_v}}) W_l(\vec{\mathcal{X}}) \prod_{v=1}^{u} (\xi_{k_v}). \tag{4.8}$$

4.4 Simplification for post-transcriptional regulation of Colicin release

So far, the analysis holds true for all systems that can be cast into the form defined in section 4.1. Let us now turn to our specific model of post-transcriptional regulation of Colicin release. With the reactions defined in chapter 1.7 we find:

$$\mathcal{A} = \begin{pmatrix} 1 & -1 & 0 & 0 & 0 & 0 & -1 & -1 & 0 & 0 \\ 0 & 0 & 1 & -1 & 0 & 0 & -1 & 0 & -1 & 0 \\ 0 & 0 & 0 & 0 & 1 & -1 & 0 & 0 & -1 & -1 \end{pmatrix} \tag{4.9}$$

$$\begin{aligned}\vec{W}(M,A,S) = (&\alpha_M, \delta_M M, \alpha_A, \delta_A A, \alpha_S, \delta_S S, k_M p_M MA, \\ &k_M(1-p_M)MA, k_S p_S AS, k_S(1-p_S)AS)^T\end{aligned} \tag{4.10}$$

From equation (4.10) we see that all derivatives higher than second order must vanish, which simplifies the sum in (4.8) significantly, i.e. u can take the values $0, 1$ or 2.

4.5 Calculation of moments

With equation (4.6) we are in the position to calculate the moments of the random variable $\vec{\xi}$. We do so by integrating equation (4.6) multiplied by the random variables whose moment is calculated by parts. Terms containing the expression $\prod_{j=1}^{i}(\partial\xi_{k_j})$ have to be integrated by parts i times. After integration, only a few terms are non-zero. For the first two moments we obtain:

$$\begin{aligned}\partial_t \langle\langle \xi_b \rangle\rangle &= \int_{-\infty}^{\infty} d\xi_1 d\xi_2 \dots d\xi_n \partial_t \pi(\vec{\xi}, t)\xi_b = \\ &= a_{bl} \sum_{u=1}^{\infty} \frac{1}{u!} \prod_{v=1}^{u} (\partial_{\mathcal{X}_{k_v}}) W_l(\vec{\mathcal{X}}) \langle\langle \prod_{v=1}^{u} (\xi_{k_v}) \rangle\rangle \end{aligned} \tag{4.11}$$

$$\partial_t \underbrace{\langle\langle \xi_b \xi_c \rangle\rangle}_{\text{symm in } b,c} = \int_{-\infty}^{\infty} d\xi_1 d\xi_2 \dots d\xi_n \partial_t \pi(\vec{\xi}, t)\xi_b\xi_c =$$

$$= a_{bl} \sum_{u=1}^{\infty} \frac{1}{u!} \prod_{v=1}^{u} (\partial_{\mathcal{X}_{k_v}}) W_l(\vec{\mathcal{X}}) \langle\langle \prod_{v=1}^{u} (\xi_{k_v}) \xi_c \rangle\rangle \tag{4.12}$$

$$+ a_{cl} \sum_{u=1}^{\infty} \frac{1}{u!} \prod_{v=1}^{u} (\partial_{\mathcal{X}_{k_v}}) W_l(\vec{\mathcal{X}}) \langle\langle \prod_{v=1}^{u} (\xi_{k_v}) \xi_b \rangle\rangle \tag{4.13}$$

$$+ a_{bl} a_{cl} \sum_{u=0}^{\infty} \frac{1}{u!} \prod_{v=1}^{u} (\partial_{\mathcal{X}_{k_v}}) W_l(\vec{\mathcal{X}}) \langle\langle \prod_{v=1}^{u} (\xi_{k_v}) \rangle\rangle \tag{4.14}$$

To calculate the third moments, we will need the following relations:

$$\partial_{\xi_i}(\xi_b\xi_c\xi_d) = \delta_{ib}\xi_c\xi_d + \delta_{ic}\xi_b\xi_d + \delta_{id}\xi_b\xi_c \tag{4.15}$$

$$\begin{aligned}\prod_{j=1}^{2}(\partial\xi_{k_j})(\xi_b\xi_c\xi_d) = {} & \xi_b(\delta_{k_1c}\delta_{k_2d} + \delta_{k_2c}\delta_{k_1d}) + \xi_c(\delta_{k_1b}\delta_{k_2d} + \delta_{k_2b}\delta_{k_1d}) \\ & + \xi_d(\delta_{k_1b}\delta_{k_2c} + \delta_{k_2b}\delta_{k_1c})\end{aligned} \tag{4.16}$$

$$\prod_{j=1}^{3}(\partial\xi_{k_j})(\xi_b\xi_c\xi_d) = \sum_{\hat{\mathrm{P}}(\mathrm{b,c,d})} \delta_{k_1b}\delta_{k_2c}\delta_{k_3d} \tag{4.17}$$

$$\prod_{j=1}^{4}(\partial\xi_{k_j})(\xi_b\xi_c\xi_d) = 0 \tag{4.18}$$

The operator $\hat{P}(b,c,d)$ signifies all permutations in (b,c,d). It follows:

$$\begin{aligned}
\partial_t \underbrace{\ll \xi_b\xi_c\xi_d \gg}_{\text{symm in } b,c,d} &= \int_{-\infty}^{\infty} d\xi_1 d\xi_2 \dots d\xi_n \partial_t \pi(\vec{\xi},t)\xi_b\xi_c\xi_d \\
&= -\int_{-\infty}^{\infty} d\vec{\xi}\partial_{\xi_i}\left(\left[a_{il}W_l(\vec{\mathcal{X}}+\vec{\xi}) - a_{il}W_l(\vec{\mathcal{X}})\right]\pi(\vec{\xi},t)\right)\xi_b\xi_c\xi_d \\
&\quad + \int_{-\infty}^{\infty} d\vec{\xi}\sum_{i=2}^{\infty}\left(\frac{(-1)^i}{i!}\prod_{j=1}^{i}(\partial\xi_{k_j})\left[\prod_{j=1}^{i}(a_{k_jl})W_l(\vec{\mathcal{X}}+\vec{\xi})\pi(\vec{\xi},t)\right]\right)\xi_b\xi_c\xi_d \\
&\overset{PI}{=} \int_{-\infty}^{\infty} d\vec{\xi}\partial_{\xi_i}(\xi_b\xi_c\xi_d)\left(\left[a_{il}W_l(\vec{\mathcal{X}}+\vec{\xi}) - a_{il}W_l(\vec{\mathcal{X}})\right]\pi(\vec{\xi},t)\right) \\
&\quad + \int_{-\infty}^{\infty} d\vec{\xi}\prod_{j=1}^{2}(\partial\xi_{k_j})(\xi_b\xi_c\xi_d)\left(\frac{1}{2}\underbrace{\prod_{j=1}^{2}(a_{k_jl})}_{\text{symm in } k_1,k_2} W_l(\vec{\mathcal{X}}+\vec{\xi})\pi(\vec{\xi},t)\right) \\
&\quad + \int_{-\infty}^{\infty} d\vec{\xi}\prod_{j=1}^{3}(\partial\xi_{k_j})(\xi_b\xi_c\xi_d)\left(\frac{1}{6}\underbrace{\prod_{j=1}^{3}(a_{k_jl})}_{\text{symm in } k_1,k_2,k_3} W_l(\vec{\mathcal{X}}+\vec{\xi})\pi(\vec{\xi},t)\right) \\
&\quad + \int_{-\infty}^{\infty} d\vec{\xi}\prod_{j=1}^{4}(\partial\xi_{k_j})(\xi_b\xi_c\xi_d) \\
&\qquad \sum_{i=4}^{\infty}\left(\frac{(-1)^i}{i!}\prod_{j=5}^{i}(\partial\xi_{k_j})\left[\prod_{j=1}^{i}(a_{k_jl})W_l(\vec{\mathcal{X}}+\vec{\xi})\pi(\vec{\xi},t)\right]\right)
\end{aligned}$$

$$= a_{bl}\sum_{u=1}^{\infty}\frac{1}{u!}\prod_{v=1}^{u}(\partial_{\mathcal{X}_{k_v}})W_l(\vec{\mathcal{X}}) \ll \prod_{v=1}^{u}(\xi_{k_v})\xi_c\xi_d \gg \tag{4.19}$$

$$+ a_{cl}\sum_{u=1}^{\infty}\frac{1}{u!}\prod_{v=1}^{u}(\partial_{\mathcal{X}_{k_v}})W_l(\vec{\mathcal{X}}) \ll \prod_{v=1}^{u}(\xi_{k_v})\xi_b\xi_d \gg \tag{4.20}$$

$$+ a_{dl}\sum_{u=1}^{\infty}\frac{1}{u!}\prod_{v=1}^{u}(\partial_{\mathcal{X}_{k_v}})W_l(\vec{\mathcal{X}}) \ll \prod_{v=1}^{u}(\xi_{k_v})\xi_b\xi_c \gg \tag{4.21}$$

$$+ a_{cl}a_{dl}\sum_{u=0}^{\infty}\frac{1}{u!}\prod_{v=1}^{u}(\partial_{\mathcal{X}_{k_v}})W_l(\vec{\mathcal{X}}) \ll \prod_{v=1}^{u}(\xi_{k_v})\xi_b \gg \tag{4.22}$$

$$+ a_{bl}a_{dl}\sum_{u=0}^{\infty}\frac{1}{u!}\prod_{v=1}^{u}(\partial_{\mathcal{X}_{k_v}})W_l(\vec{\mathcal{X}}) \ll \prod_{v=1}^{u}(\xi_{k_v})\xi_c \gg \tag{4.23}$$

$$+ a_{bl}a_{cl}\sum_{u=0}^{\infty}\frac{1}{u!}\prod_{v=1}^{u}(\partial_{\mathcal{X}_{k_v}})W_l(\vec{\mathcal{X}}) \ll \prod_{v=1}^{u}(\xi_{k_v})\xi_d \gg \tag{4.24}$$

$$+ a_{bl}a_{cl}a_{dl}\sum_{u=0}^{\infty}\frac{1}{u!}\prod_{v=1}^{u}(\partial_{\mathcal{X}_{k_v}})W_l(\vec{\mathcal{X}}) \ll \prod_{v=1}^{u}(\xi_{k_v}) \gg \tag{4.25}$$

The same steps of calculation can be applied to the fourth moment as well. To avoid too longish expressions, we just state the result:

$$\partial_t \underbrace{\ll \xi_b\xi_c\xi_d\xi_e \gg}_{\text{symm in } b,c,d,e} = \int_{-\infty}^{\infty} d\xi_1 d\xi_2 \dots d\xi_n \partial_t \pi(\vec{\xi},t)\xi_b\xi_c\xi_d\xi_e$$

$$= a_{bl}\sum_{u=1}^{\infty}\frac{1}{u!}\prod_{v=1}^{u}(\partial_{\mathcal{X}_{k_v}})W_l(\vec{\mathcal{X}}) \ll \prod_{v=1}^{u}(\xi_{k_v})\xi_c\xi_d\xi_e \gg \tag{4.26}$$

$$+ a_{cl}\sum_{u=1}^{\infty}\frac{1}{u!}\prod_{v=1}^{u}(\partial_{\mathcal{X}_{k_v}})W_l(\vec{\mathcal{X}}) \ll \prod_{v=1}^{u}(\xi_{k_v})\xi_b\xi_c\xi_d \gg \tag{4.27}$$

$$+ a_{dl}\sum_{u=1}^{\infty}\frac{1}{u!}\prod_{v=1}^{u}(\partial_{\mathcal{X}_{k_v}})W_l(\vec{\mathcal{X}}) \ll \prod_{v=1}^{u}(\xi_{k_v})\xi_b\xi_c\xi_e \gg \tag{4.28}$$

$$+ a_{el}\sum_{u=1}^{\infty}\frac{1}{u!}\prod_{v=1}^{u}(\partial_{\mathcal{X}_{k_v}})W_l(\vec{\mathcal{X}}) \ll \prod_{v=1}^{u}(\xi_{k_v})\xi_b\xi_c\xi_d \gg \tag{4.29}$$

$$+ a_{bl}a_{cl}\sum_{u=0}^{\infty}\frac{1}{u!}\prod_{v=1}^{u}(\partial_{\mathcal{X}_{k_v}})W_l(\vec{\mathcal{X}}) \ll \prod_{v=1}^{u}(\xi_{k_v})\xi_d\xi_e \gg \tag{4.30}$$

$$+ a_{bl}a_{dl}\sum_{u=0}^{\infty}\frac{1}{u!}\prod_{v=1}^{u}(\partial_{\mathcal{X}_{k_v}})W_l(\vec{\mathcal{X}}) \ll \prod_{v=1}^{u}(\xi_{k_v})\xi_c\xi_e \gg \tag{4.31}$$

$$+ a_{bl}a_{el}\sum_{u=0}^{\infty}\frac{1}{u!}\prod_{v=1}^{u}(\partial_{\mathcal{X}_{k_v}})W_l(\vec{\mathcal{X}}) \ll \prod_{v=1}^{u}(\xi_{k_v})\xi_c\xi_d \gg \tag{4.32}$$

$$+ a_{cl}a_{dl}\sum_{u=0}^{\infty}\frac{1}{u!}\prod_{v=1}^{u}(\partial_{\mathcal{X}_{k_v}})W_l(\vec{\mathcal{X}}) \ll \prod_{v=1}^{u}(\xi_{k_v})\xi_b\xi_e \gg \tag{4.33}$$

$$+ a_{cl}a_{el}\sum_{u=0}^{\infty}\frac{1}{u!}\prod_{v=1}^{u}(\partial_{\mathcal{X}_{k_v}})W_l(\vec{\mathcal{X}}) \ll \prod_{v=1}^{u}(\xi_{k_v})\xi_b\xi_d \gg \tag{4.34}$$

$$+ a_{dl}a_{el}\sum_{u=0}^{\infty}\frac{1}{u!}\prod_{v=1}^{u}(\partial_{\mathcal{X}_{k_v}})W_l(\vec{\mathcal{X}}) \ll \prod_{v=1}^{u}(\xi_{k_v})\xi_b\xi_c \gg \tag{4.35}$$

$$+ a_{bl}a_{cl}a_{dl}\sum_{u=0}^{\infty}\frac{1}{u!}\prod_{v=1}^{u}(\partial_{\mathcal{X}_{k_v}})W_l(\vec{\mathcal{X}}) \ll \prod_{v=1}^{u}(\xi_{k_v})\xi_e \gg \tag{4.36}$$

$$+ a_{bl}a_{cl}a_{el}\sum_{u=0}^{\infty}\frac{1}{u!}\prod_{v=1}^{u}(\partial_{\mathcal{X}_{k_v}})W_l(\vec{\mathcal{X}}) \ll \prod_{v=1}^{u}(\xi_{k_v})\xi_d \gg \tag{4.37}$$

$$+ a_{bl}a_{dl}a_{el}\sum_{u=0}^{\infty}\frac{1}{u!}\prod_{v=1}^{u}(\partial_{\mathcal{X}_{k_v}})W_l(\vec{\mathcal{X}}) \ll \prod_{v=1}^{u}(\xi_{k_v})\xi_c \gg \tag{4.38}$$

$$+ a_{cl}a_{dl}a_{el}\sum_{u=0}^{\infty}\frac{1}{u!}\prod_{v=1}^{u}(\partial_{\mathcal{X}_{k_v}})W_l(\vec{\mathcal{X}}) \ll \prod_{v=1}^{u}(\xi_{k_v})\xi_b \gg \tag{4.39}$$

$$+ a_{bl}a_{cl}a_{dl}a_{el}\sum_{u=0}^{\infty}\frac{1}{u!}\prod_{v=1}^{u}(\partial_{\mathcal{X}_{k_v}})W_l(\vec{\mathcal{X}}) \ll \prod_{v=1}^{u}(\xi_{k_v}) \gg \tag{4.40}$$

4.6 Scaling of terms in the equations of moments

We have neglected the system size parameter Ω so far, which in fact seems odd, since van Kampen's expansion is also known as the Ω-expansion. There are two important points why we have followed this procedure:

1. It is indeed possible to find a parameter Ω that fulfills the requirement needed for an Ω-expansion, namely $(\dot{M},\dot{A},\dot{S})^T = \vec{F}(M,A,S) = \Omega\vec{f}(M/\Omega, A/\Omega, S/\Omega)$. Ω has to be a large quantity proportional to the system size. However, inserting real parameters, we find $\Omega \ll 1$. We conclude, that the parameter Ω is not well-defined in our model, but would be only an artificial construct.
2. When looking at the scaling properties of $\langle\langle\xi\rangle\rangle, \langle\langle\xi^2\rangle\rangle, \ldots$ we cannot simply group all terms of the same order in $\sqrt{\Omega}^i$, $i \in \mathbf{N}$, since all of these terms come in combination with $\langle\langle\xi\rangle\rangle, \langle\langle\xi^2\rangle\rangle, \ldots$ terms which in turn have a specific scaling property, as we shall see.

In the following, we define the system size parameter $\tilde{\Omega}$ as the total number of molecules present in the system. With this definition we will work out the scaling properties of $\langle\langle\xi\rangle\rangle$, $\langle\langle\xi^2\rangle\rangle$, $\langle\langle\xi^3\rangle\rangle$ and $\langle\langle\xi^4\rangle\rangle$ in the system size parameter $\tilde{\Omega}$ as well as the significance of each term in the equations for the moments.

Scaling of specific terms:

- The stoichiometric matrix A_{bl} scales with $\mathcal{O}(1)$.
- The reaction rate matrix is quadratic in the molecule numbers, such that $\vec{W}(\vec{\mathcal{X}})$ scales with $\mathcal{O}(\tilde{\Omega}^2)$.
- Each derivative with respect to $\mathcal{X}_i$ introduces a factor $\mathcal{O}(1/\tilde{\Omega})$.

The equations derived for the moments (see section 4.5) may be classified into terms with equal scaling behavior. The scaling behavior depends on u $(0, 1$ or $2)$ and the order of the moment. Since we are interested in the stationary values of fluctuations, we can set all derivatives with respect to time equal to zero, and find:

Equations	u=0	u=1	u=2
(4.11)	-	$\mathcal{O}(\tilde{\Omega})\langle\langle\xi\rangle\rangle$	$\mathcal{O}(1)\langle\langle\xi^2\rangle\rangle$

Table 4.1. Scaling of terms for first moments

Equations	u=0	u=1	u=2
(4.12)-(4.13)	-	$\mathcal{O}(\tilde{\Omega})\langle\langle\xi^2\rangle\rangle$	$\mathcal{O}(1)\langle\langle\xi^3\rangle\rangle$
(4.14)	$\mathcal{O}(\tilde{\Omega}^2)$	$\mathcal{O}(\tilde{\Omega})\langle\langle\xi\rangle\rangle$	$\mathcal{O}(1)\langle\langle\xi^2\rangle\rangle$

Table 4.2. Scaling of terms for second moments

Equations	u=0	u=1	u=2
(4.19)-(4.21)	-	$\mathcal{O}(\tilde{\Omega})\langle\langle\xi^3\rangle\rangle$	$\mathcal{O}(1)\langle\langle\xi^4\rangle\rangle$
(4.22)-(4.24)	$\mathcal{O}(\tilde{\Omega}^2)\langle\langle\xi\rangle\rangle$	$\mathcal{O}(\tilde{\Omega})\langle\langle\xi^2\rangle\rangle$	$\mathcal{O}(1)\langle\langle\xi^3\rangle\rangle$
(4.25)	$\mathcal{O}(\tilde{\Omega}^2)$	$\mathcal{O}(\tilde{\Omega})\langle\langle\xi\rangle\rangle$	$\mathcal{O}(1)\langle\langle\xi^2\rangle\rangle$

Table 4.3. Scaling of terms for third moments

Equations	u=0	u=1	u=2
(4.26)-(4.29)	-	$\mathcal{O}(\tilde{\Omega})\langle\langle\xi^4\rangle\rangle$	$\mathcal{O}(1)\langle\langle\xi^5\rangle\rangle$
(4.30)-(4.35)	$\mathcal{O}(\tilde{\Omega}^2)\langle\langle\xi^2\rangle\rangle$	$\mathcal{O}(\tilde{\Omega})\langle\langle\xi^3\rangle\rangle$	$\mathcal{O}(1)\langle\langle\xi^4\rangle\rangle$
(4.36)-(4.39)	$\mathcal{O}(\tilde{\Omega}^2)\langle\langle\xi\rangle\rangle$	$\mathcal{O}(\tilde{\Omega})\langle\langle\xi^2\rangle\rangle$	$\mathcal{O}(1)\langle\langle\xi^3\rangle\rangle$
(4.40)	$\mathcal{O}(\tilde{\Omega}^2)$	$\mathcal{O}(\tilde{\Omega})\langle\langle\xi\rangle\rangle$	$\mathcal{O}(1)\langle\langle\xi^2\rangle\rangle$

Table 4.4. Scaling of terms for fourth moments

Looking at the dominant terms in the tables 4.1, 4.2, 4.3 and 4.4, we deduce:

$$\langle\langle\xi\rangle\rangle = \mathcal{O}(1/\tilde{\Omega})\langle\langle\xi^2\rangle\rangle \tag{4.41}$$

$$\langle\langle\xi^2\rangle\rangle = \mathcal{O}(\tilde{\Omega}) + \mathcal{O}(1/\tilde{\Omega})\langle\langle\xi^3\rangle\rangle \tag{4.42}$$

$$\langle\langle\xi^3\rangle\rangle = \mathcal{O}(\tilde{\Omega}) + \mathcal{O}(1)\langle\langle\xi^2\rangle\rangle + \mathcal{O}(1/\tilde{\Omega})\langle\langle\xi^4\rangle\rangle \tag{4.43}$$

$$\langle\langle\xi^4\rangle\rangle = \mathcal{O}(\tilde{\Omega})\langle\langle\xi^2\rangle\rangle + \mathcal{O}(1/\tilde{\Omega})\langle\langle\xi^5\rangle\rangle \tag{4.44}$$

Since van Kampen's expansion is only valid for small noise, we expect ξ to be of order $\mathcal{O}\sqrt(\tilde{\Omega})$ and thus $\langle\langle\xi^5\rangle\rangle$ is smaller than of order $\mathcal{O}(\tilde{\Omega}^3)$.

Hence, we find: $\boxed{\langle\langle\xi\rangle\rangle = \mathcal{O}(1),\ \langle\langle\xi^2\rangle\rangle = \mathcal{O}(\tilde{\Omega}),\ \langle\langle\xi^3\rangle\rangle = \mathcal{O}(\tilde{\Omega}),\ \langle\langle\xi^4\rangle\rangle = \mathcal{O}(\tilde{\Omega}^2)}$.

Using this result, we marked all terms in the tables above of order $\mathcal{O}(\tilde{\Omega}^3)$ in green, all terms of order $\mathcal{O}(\tilde{\Omega}^2)$ in blue and all terms of order $\mathcal{O}(\tilde{\Omega})$ in red. In each of the tables 4.1, 4.2, 4.3 and 4.4, we will call the dominant terms first order terms, followed by second order terms that are of order $\mathcal{O}(\tilde{\Omega})$ smaller than first order terms. Consequently, third order terms are of order $\mathcal{O}(\tilde{\Omega}^2)$ smaller than first order terms and so on.

4.7 Calculation of the Fano factor of long mRNA

The scaling behavior of all terms that are necessary to calculate fluctuations, i.e. second moments, are given in table 4.2. The two dominating terms are marked blue. Thus, in first order it is sufficient to take only these two terms into account. This procedure is in fact the standard procedure used in the literature [4, 28, 29]. Due to nonlinear reaction rates, we get $u = 2$ terms, which mediate the coupling to higher moments. If we want to calculate fluctuations to higher than just first order, we have to take into consideration both red and blue terms in table 4.2. It follows that we have to include first moments (table 4.1) and third moments (table 4.3). First moments are simple to implement, because they couple only to second moments. To calculate third moments, however, we have to consider all dominant terms (blue) in table 4.3. Unfortunately, these terms include also fourth moments, which we would have to calculate via the green terms in table 4.4.

Hence, if we want to consider higher order terms in our calculation of fluctuations, we have to either work out the coupled equations from first moments up to fourth

moments, or truncate the coupled equations by introducing a suitable closure-relation. Furthermore, in the threshold region, where the coupling has its largest influence, copy numbers are pretty low, which could bias all order arguments we have used so far. Nonetheless, we would like to test how well the calculated results fit to data obtained by Gillespie simulations. To this end we started from first order calculations and work our way up to higher order calculations.

We began with the standard procedure by considering only the two dominant terms (blue) in table 4.2. Comparing with the result of Gillespie simulations (see S3 Fig) shows an adequate match, which overestimates fluctuations in the vicinity of the threshold. When studying different parameter sets, it can be seen that, although fluctuations are overestimated, the shape of the surface is well matched. We continued and included higher moments, both by an adequate closure relation after the second moment, as well as by actually implementing all terms of table 4.1, all terms of 4.2, all blue terms of table 4.3 and all green terms of 4.4. However, the results for these methods were (in general) worse than those from considering the dominant terms only. Thus, we chose the first order method to calculate the Fano factor.

Bibliography

1. Clark DP, Pazdernik NJ. Molecular biology: Understanding the genetic revolution. ACACL; 2012.

2. Suzuki K, Babitzke P, Kushner SR, Romeo T. Identification of a novel regulatory protein (CsrD) that targets the global regulatory RNAs CsrB and CsrC for degradation by RNase E. Genes & development. 2006;20(18):2605.

3. Kærn M, Elston TC, Blake WJ, Collins JJ. Stochasticity in gene expression: from theories to phenotypes. Nature Reviews Genetics. 2005;6(6):451–464.

4. Gardiner CW. Stochastic methods. Springer; 2009.

5. Levine E, Zhang Z, Kuhlman T, Hwa T. Quantitative characteristics of gene regulation by small RNA. PLoS biology. 2007;5(9):e229.

6. Legewie S, Dienst D, Wilde A, Herzel H, Axmann IM. Small RNAs establish delays and temporal thresholds in gene expression. Biophysical journal. 2008;95(7):3232–3238.

7. Taniguchi Y, Choi PJ, Li GW, Chen H, Babu M, Hearn J, et al. Quantifying E. coli Proteome and Transcriptome with Single-Molecule Sensitivity in Single Cells. Science (80-). 2010;329.

8. Gudapaty S, Suzuki K, Wang X, Romeo T, Wang XIN, Babitzke P. Regulatory Interactions of Csr Components : the RNA Binding Protein CsrA Activates csrB Transcription in Escherichia coli Regulatory Interactions of Csr Components : the RNA Binding Protein CsrA Activates csrB Transcription in Escherichia coli. J Bacteriol. 2001;.

9. Legewie S, Dienst D, Wilde A, Herzel H, Axmann IM. Small RNAs establish delays and temporal thresholds in gene expression. Biophys J. 2008;95(7):3232–3238. Available from: `http://dx.doi.org/10.1529/biophysj.108.133819`.

10. Vakulskas CA, Leng Y, Abe H, Amaki T, Okayama A, Babitzke P, et al. Antagonistic control of the turnover pathway for the global regulatory sRNA CsrB by the CsrA and CsrD proteins. 2016;p. 1–15.

11. Leng Y, Vakulskas CA, Zere TR, Pickering BS, Watnick PI, Babitzke P, et al. Regulation of CsrB/C sRNA decay by EIIA. 2016;(2015):627–639.

12. Eppler T, Postma P, Boos W. Glycerol-3-Phosphate-Induced Catabolite Repression in. 2002;184(11):3044–3052.

13. Adamson DN, Lim HN. Rapid and robust signaling in the CsrA cascade via RNA–protein interactions and feedback regulation. Proc Natl Acad Sci. 2013;110(32):13120–13125. Available from: http://www.pnas.org/content/110/32/13120.abstract.

14. Yang TY, Sung YM, Lei GS, Romeo T, Chak KF. Posttranscriptional repression of the cel gene of the ColE7 operon by the RNA-binding protein CsrA of Escherichia coli. Nucleic acids research. 2010;38(12):3936–3951.

15. Nath K, Koch AL. Protein Degradation in Escherichia coli I. MEASUREMENT OF RAPIDLY AND SLOWLY DECAYING COMPONENTS. Journal of Biological Chemistry. 1970;245(11):2889–2900.

16. Nath K, Koch A, et al. Protein degradation in Escherichia coli. II. Strain differences in the degradation of protein and nucleic acid resulting from starvation. The Journal of biological chemistry. 1971;246(22):6956.

17. Dubey AK, Baker CS, Romeo T, Babitzke P. CsrA - RNA interaction RNA sequence and secondary structure participate in high-affinity CsrA – RNA interaction. Rna. 2005;(11):1579–1587.

18. Mercante J, Edwards AN, Dubey AK, Babitzke P, Romeo T. Molecular Geometry of CsrA (RsmA) Binding to RNA and Its Implications for Regulated Expression. J Mol Biol. 2009;392(2):511–528. Available from: http://dx.doi.org/10.1016/j.jmb.2009.07.034.

19. Yang TY, Sung YM, Lei GS, Romeo T, Chak KF. Posttranscriptional repression of the cel gene of the ColE7 operon by the RNA-binding protein CsrA of Escherichia coli. Nucleic Acids Res. 2010;38(12):3936–3951.

20. Mercante J, Edwards AN, Dubey AK, Babitzke P. Implications for Regulated Expression. 2010;392(2):511–528.

21. E KH. Cell volume increase in Escherichia coli after shifts to richer media. The Journal of Bacteriology. 1990;72(1):94–101.

22. Romeo T, et al. Global regulation by the small RNA-binding protein CsrA and the non-coding RNA molecule CsrB. Molecular microbiology. 1998;29(6):1321.

23. EcoCyc. EcoCyc - A member of the BioCyc database collection;. Available online at http://ecocyc.org.

24. Shimoni Y, Altuvia S, Margalit H, Biham O. Stochastic analysis of the SOS response in Escherichia coli. PloS one. 2009;4(5):e5363.

25. Gillespie DT. Exact stochastic simulation of coupled chemical reactions. The journal of physical chemistry. 1977;81(25):2340–2361.

26. Cascales E, Buchanan SK, Duché D, Kleanthous C, Lloubes R, Postle K, et al. Colicin biology. Microbiology and Molecular Biology Reviews. 2007;71(1):158–229.

27. Bionumbers. RNA polymerase transcription rate of stable RNA - BNID 100060; 2008. Available online at http://bionumbers.hms.harvard.edu/bionumber.aspx?&id=100060&ver=23. Website.

28. Van Kampen N. Stochastic Processes in Physics and Chemistry. North-Holland; 2007.

29. Tsimring L, Volfson D, Hasty J. Stochastically driven genetic circuits. Chaos: An Interdisciplinary Journal of Nonlinear Science. 2006;16(2):026103–026103.
